# Characterization of human CD34^+^ HSPC-derived neutrophils with limited myeloid-derived immunosuppressive cell activity

**DOI:** 10.1016/j.isci.2025.113404

**Published:** 2025-08-20

**Authors:** Steven D.S. Webbers, Angela A.F. Gankema, Fleur van Oosterom, Felipe Rojas-Rodriguez, Dané S. Koops, Anna K. Klaus, Judy Geissler, Teunis J.P. van Dam, Hanke L. Matlung, Robin van Bruggen, Helin Tercan, Arie J. Hoogendijk, Taco W. Kuijpers

**Affiliations:** 1 Department of Molecular Hematology, Sanquin Research, University of Amsterdam, 1066 CX Amsterdam, the Netherlands; 2 Department of Pediatric Immunology, Rheumatology and Infectious Disease, Emma Children’s Hospital Amsterdam University Medical Center (Amsterdam UMC), University of Amsterdam, 1105 AZ Amsterdam, the Netherlands

**Keywords:** Immunology, Proteomics, Transcriptomics

## Abstract

Neutrophils have limited utility as a fundamental research model and transfusion product due to their short lifespan. Here, we cultured CD34^+^ hematopoietic stem and progenitor cell (HSPC)-derived neutrophils and compared them to peripheral blood neutrophils in terms of morphology, phenotype, and function. Our culture system resulted in morphologically mature CD15^+^CD11b^+^CD16^high^ neutrophils with effector functions almost indistinguishable from blood neutrophils, confirmed by a high similarity in transcription and protein abundance patterns. While exhibiting microbial killing capacity and antibody-dependent cellular cytotoxicity, these cells were deficient in myeloid-derived suppressor cell activity. This deficiency in immunosuppressive activity correlated with a distinct granular composition in comparison to blood neutrophils, instead of immunosuppressive characteristics that are currently held to define neutrophil-MDSC phenotype. Taken together, our cultured neutrophils closely resemble blood neutrophils, offering a repository for fundamental research and a step toward an effective transfusion product with limited immunosuppressive activity as a functional property.

## Highlights

• Culturing CD34^+^ HSPCs in SL-II medium results in mature and functional neutrophils
• Cultured neutrophils are only deficient in immunosuppressive activity
• Using multi-omics, cultured neutrophils show a distinct granular composition

## Graphical abstract

**Figure:**
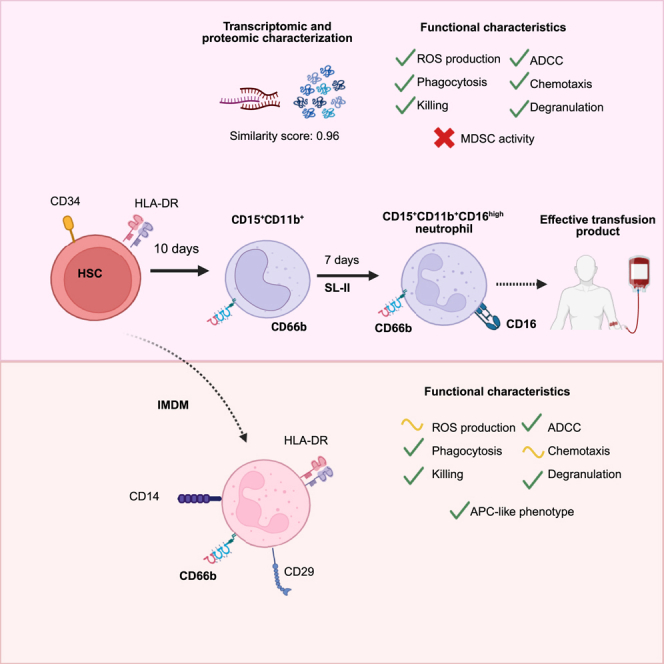

## Introduction

Neutrophils comprise 60–70% of all circulating leukocytes in humans and are the first responders at sites of infection.^1^ Also known as polymorphonuclear cells (PMNs), neutrophils fulfill a key role in host defense by clearing bacterial and fungal pathogens through their phagocytic capacity, the production of reactive oxygen species (ROS), neutrophil extracellular trap (NET) formation,^1^^,^^2^^,^^3^ and release of granules with toxic proteins.^4^^,^^5^ Neutrophils originate in the bone marrow from CD34^+^ hematopoietic stem and progenitor cells (HSPCs) during a process called granulopoiesis.^4^^,^^6^ The release of mature neutrophils from the proliferative myeloblast takes around 14 days. In the course of their differentiation into non-cycling end-stage neutrophils, PMNs develop these anti-microbial functions, lose their proliferative potential, and adapt their metabolic activity. Once egressed from the bone marrow, these cells circulate only for a short while before entering into the tissues, where they reside for one or two days. Conditions leading to low neutrophil counts, called neutropenia, can result in life-threatening recurrent bacterial and fungal infections.^6^^,^^7^^,^^8^^,^^9^^,^^10^ Neutrophils also play a role in cancer, where they can perform anti-tumor activity by antibody-dependent cellular cytotoxicity (ADCC).^11^^,^^12^^,^^13^ However, neutrophils are mostly considered to exert pro-tumorigenic activity by myeloid-derived suppressor cell (MDSC) activity against T cells directed against the tumor cells.^11^^,^^14^ An increased neutrophil-to-lymphocyte ratio in blood, as well as an increased number of neutrophils in the tumor microenvironment itself, are typically associated with poor prognosis.^9^^,^^15^ Both neutrophil ADCC and MDSC activity are dependent on β2 integrin mediated cell-cell contact and trogocytosis, while MDSC activity also requires ROS production and degranulation.^14^^,^^16^ Nonetheless, the exact molecular mechanisms behind these processes and whether these pro- and anti-tumorigenic activities are molecularly similar have remained largely unknown.^9^

Studies on the molecular mechanisms behind neutrophil functionality are impeded by their short life span, donor variation in primary human material and poor resemblance of cell lines (e.g., HL-60) and mouse neutrophils to circulating human neutrophils.^5^^,^^17^^,^^18^^,^^19^ Moreover, studying human neutrophil behavior by using patient material is difficult due to the low prevalence of genetic aberrations, and these phenotypes are poorly recapitulated by the use of chemical inhibitors. The neutrophil research field would therefore benefit from appropriate cellular model systems in which cells could be studied and genetically modified.^6^^,^^20^ Current stem cell-derived neutrophil culture protocols generally fall short to recapitulate terminally differentiated blood neutrophils.^20^^,^^21^^,^^22^^,^^23^^,^^24^ Here, we describe an improved serum-free culture system that results in late-stage non-proliferative neutrophils derived from CD34^+^ HSPCs. By phenotypical characterization, we obtained neutrophils characterized by CD15^+^CD11b^+^CD66b^+^CD16^high^ expression. Using mass spectrometry-based proteomics and transcriptomics (Bulk RNA-seq), these cultured neutrophils were found to closely resemble mature blood neutrophils. Although functionally similar in their classical effector functions, these cultured neutrophils were found to be deficient in MDSC activity. This deficiency in immunosuppressive activity correlates with a distinct granular content compared to PMNs. Taken together, we obtained neutrophils showing similar molecular features and functional host defense characteristics to those observed in PMNs enabling further in-depth molecular studies and serve as a scaffold for the development of neutrophil-based cellular therapies.

## Results

### CD34^+^ hematopoietic stem and progenitor cell-derived neutrophils closely resemble blood neutrophils

**Figure 1:**
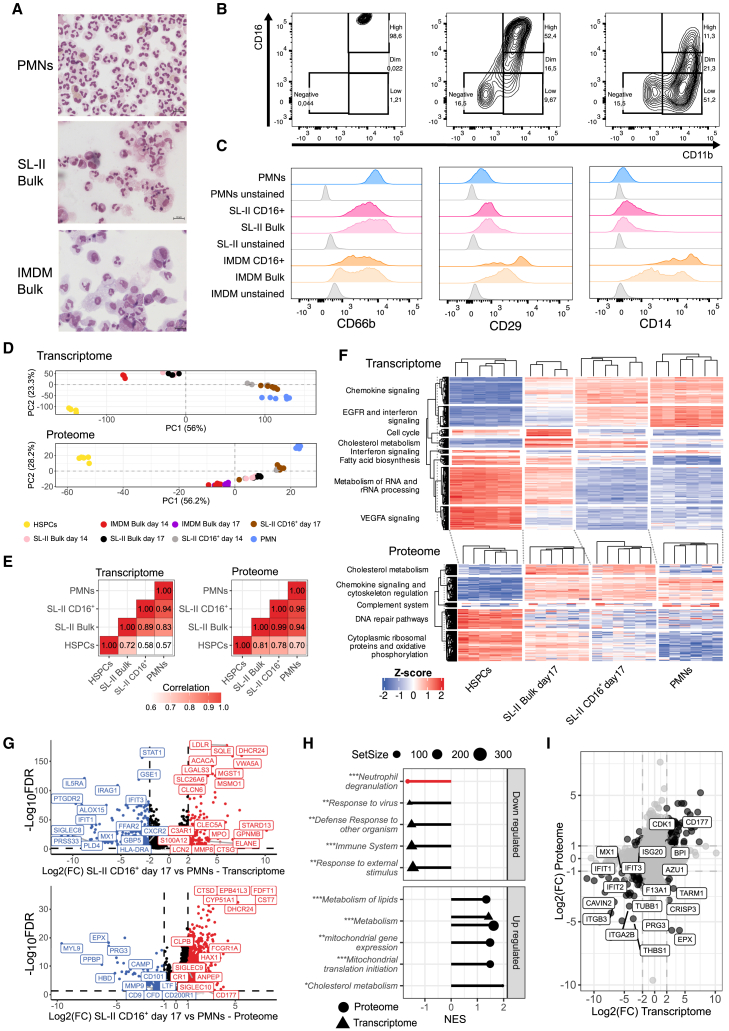
CD34^+^ HSPC-derived neutrophils cultured in SL-II medium showed higher similarity to PMNs compared to CD34^+^ HSPC-derived neutrophils cultured in IMDM (A) Representative cytospins of PMNs, CD34^+^ HSPC-derived neutrophils cultured in SL-II (Bulk) and IMDM medium (Bulk) after Giemsa-May staining. Scalebars set at 10 μm, *n* = 14. (B) Gating strategy for comparing PMNs versus CD34^+^ HSPC-derived neutrophils cultured in SL-II or IMDM based on CD11b and CD16 expression as measured by flow cytometry. (C) Representative flow cytometric analysis of surface markers CD66b, CD29 and CD14 expressed on Bulk and CD16^high^ CD34^+^ HSPC-derived neutrophils cultured in either SL-II (pink) or IMDM medium (orange) and PMNs (blue). Gray histograms show unstained controls. *n* = 8 for CD66b, *n* = 4 for CD29 and CD14. (D) PCA plot showing transcriptomes and non-imputed proteomes for CD34^+^ HSPCs (yellow), SL-II Bulk obtained at days 14 (light pink) and 17 (black), IMDM Bulk obtained at day 14 (red) and 17 (purple), SL-II fractions MACS sorted for CD16^+^ obtained at day 14 (gray) and 17 (brown), and PMNs (blue). For SL-II Bulk day 17 transcriptomics *n* = 4, otherwise *n* = 6. (E) Pearson correlation between day 17 SL-II CD16^+^ neutrophils compared to CD34^+^ HSPCs and PMNs. Correlation values range between 0.6 and 1. (F) Heatmap of z-scores for transcriptome and proteome samples obtained from day 17 SL-II Bulk neutrophils, day 17 SL-II CD16^+^ neutrophils, PMNs, and CD34^+^ HSPCs. All transcripts or proteins that were considered differentially abundance between day 0 SL-II Bulk, day 10 SL-II Bulk, day 14 SL-II CD16^+^ neutrophils and day 17 SL-II CD16^+^ neutrophils when compared to PMNs were included. (G) Volcano plots of differentially expressed transcripts and proteins between day 17 SL-II CD16^+^ neutrophils compared to PMNs. (H) Functional enrichment showing the normalized enrichment score (NES) of molecular functions that were enriched within up- or downregulated transcripts and proteins in day 17 SL-II CD16^+^ neutrophils compared to PMNs. Molecular mechanisms were obtained from different databases, including Wiki-Pathways (∗), Gene Onthology (∗∗), or Reactome (∗∗∗). Neutrophil degranulation is highlighted in red. (I) Scatterplot comparing transcriptome and proteome effect size estimates for all transcript/protein pairs that were identified when comparing day 17 SL-II CD16^+^ neutrophils and PMNs. Gene/protein pairs highlighted in black were considered statistically significant after multiple testing correction FDR <0.05 and |log2 fold change| > 1 for proteome and 2 for transcriptome. *n* values represent the number of individual donor samples.

To assess the terminal differentiation of CD34^+^ HSPC-derived neutrophils in culture, we compared late-stage fractions of CD34^+^ HSPC-derived neutrophils cultured in either Iscove’s Modified Dulbecco’s Medium (IMDM) or Stemline II (SL-II) medium to circulating neutrophils derived from blood (PMNs). Multipotent HSPCs expressing CD34 were isolated and purified from mobilized peripheral blood of donors. These CD34^+^ HSPCs were cultured using an improved culture protocol with SL-II medium (Figure S1). Morphologically, both SL-II and IMDM CD34^+^ HSPC-derived cells closely resemble the multi-lobular organization of PMN nuclei (Figure 1A). To characterize the terminal differentiation of these cultures by flow cytometry, we defined populations present in the cultures using CD15^+^ and CD11b^+^CD16^high^, as well as the expression of several differentiation markers (Figure S1). A larger fraction of CD11b^+^ and CD16^high^ cells was observed in SL-II (43,4% ± 9.5%) compared to IMDM cultures (12,8% ± 15,9%) (Figure 1B). SL-II and IMDM cultures showed similar expressions of the neutrophil-specific marker CD66b and higher expression of EMR3, while SL-II cultured neutrophils displayed much lower expression of CD14 and CD29, more closely resembling PMNs (Figure 1C; Table S1). Differentiation markers such as EMR3, CD10, CD101 and Siglec-9^9^^,^^21^^,^^25^^,^^26^ showed similar expression for SL-II cultured cells compared to PMNs (Figure S1; Table S1). This similarity was also observed measuring 20 additional surface markers (Table S1), supporting the notion that SL-II cultured cells more closely resemble PMNs in their surface marker expression compared to their IMDM counterparts.

To study neutrophil terminal differentiation at transcriptomic and proteomic level, we performed transcriptomics using Bulk RNA-seq and mass spectrometry-based proteomics on CD34^+^ HSPC-derived neutrophils cultured in both IMDM and SL-II media. For SL-II cultures, samples included Bulk cells and MACS-sorted CD16^+^ cells collected on day 14 and 17 of culture (Figure S1). Considering that only a small fraction of CD34^+^ HSPC-derived neutrophils cultured in IMDM developed in CD16^high^ cells (Figure 1B), only transcripts for Bulk samples at day 14 for this culture could be included. Principal component analysis (PCA) showed that IMDM Bulk samples differ the most from PMNs while the adjacency of SL-II CD16^+^ day 14 and day 17 culture samples to PMNs indicates greater transcriptomic and proteomic similarity (Figure 1D). Pearson correlation also indicated a strong similarity (i.e., 0.94 for transcriptome and 0.96 for proteome) between SL-II CD16^+^ cells at day 17 and PMNs (Figures 1E and S2).

To further characterize the molecular similarities between SL-II CD16^+^ and PMNs in comparison to SL-II Bulk day 17 samples, we identified differentially expressed genes and proteins in comparison to the mobilized CD34^+^ HSPCs starting material. In total, 2,360 genes (FDR <0.05 & Log2FC threshold = 2) and 4,487 proteins (FDR <0.05 & Log2FC threshold = 1) were observed to be different in at least one of these conditions (Figure 1F). Transcripts involved in RNA metabolism, VEGFA signaling and rRNA processing, and proteins related to ribosomes and oxidative phosphorylation were reduced in level for SL-II CD16^+^ similar to PMNs, and to a lesser extent in SL-II Bulk. On the proteome, SL-II Bulk, SL-II CD16^+^ and PMNs all exhibited increased chemokine signaling (e.g., PREX1, RAC2, VAV1, FGR, JAK2, RASGRP2, PAK1, CXCR2 and STAT3) and cytoskeleton regulation associated protein levels (e.g., ARPC5, WASF2, ACTB, CYFIP2 and PFN1) in comparison to HSPCs. In contrast, the abundance of transcripts associated to cell cycle, EGFR and interferon signaling in the Bulk fraction was more similar to HSPCs than the CD16^+^ fraction. Contrary to both HSPCs and PMNs, Bulk and SL-II CD16^+^ fractions at day 17 displayed lower transcript levels for interferon signaling genes (e.g., *GBP1, IFIT1, OAS3, TRIM22, IFIT5 and PLCG1*). Similarly, only PMNs showed higher protein levels involved in the complement system (e.g., C3, C3AR1 and VSIG4). Bulk and SL-II CD16^+^ fractions at day 17 displayed higher levels of proteins involved in cholesterol metabolism, which was consistent when comparing SL-II and IMDM fractions at day 14 to HSPCs and PMNs (Figure S2).

Next, we focused on the remaining differences between the day 17 SL-II CD16^+^ neutrophil fraction and PMNs. We found a total of 201 genes and 848 proteins upregulated and 295 genes and 154 proteins downregulated (FDR <0.05; Log2LFC >1 for proteomics and >2 for transcriptomics) (Figure 1G). Here we focus only on transcripts for which we have protein data available. For example, *STAT1*, *SIGLEC8* and *HLA-DRA* transcripts were downregulated, while CFD, CAMP and CD101 were downregulated at the protein level. On the contrary, at the transcript level *LDLR*, *LGALS3*, *C3AR1*, *LCN2* and *CLEC5A*, and at the protein level CD177, SIGLEC10, CTSD and CST7 were upregulated. The complete list of transcripts and proteins that were different between the SL-II CD16^+^ fraction and PMNs is specified in Table S2. To gain a more detailed understanding of the associated processes, we performed gene set enrichment analysis (*p* value <0.05) using wiki-pathways,^27^ reactome^28^ and gene ontology^29^ on up- and downregulated transcripts and protein subsets separately (Figure 1H). Transcriptomic and proteomic data showed the upregulation of metabolic and mitochondrial mechanisms in the day 17 SL-II CD16^+^ fraction compared to PMNs. Notably, a small subset of 12 proteins involved in neutrophil degranulation was found to be downregulated in SL-II CD16^+^ at day 17 (Table S3).

To determine whether there are differences due to a mismatch of protein and transcript abundance between day 17 SL-II CD16^+^ and PMNs, the correspondence in effect size estimates for transcript/protein pairs was assessed (FDR <0.05; Figure 1I). Among the 118 transcript/protein pairs that were differentially expressed in both transcriptome and proteome, 98 (83.1%) showed positive correspondence indicating an overall match between transcriptome and proteome. For example, CD177, CDK1 and BPI showed an increase in transcript/protein pairs in day 17 SL-II CD16^+^ neutrophils compared to PMNs. In contrast, transcript/protein pairs for the interferon signature (i.e., MX1, IFIT1, IFIT2 and IFIT3) displayed decreased abundance in day 17 SL-II CD16^+^ cells when compared to PMNs, which may reflect a less pro-inflammatory phenotype. A subset of six transcript/protein pairs (i.e., AZU1, CRISP3, EPX, F13A1, PRG3 and TARM1) showed positive effect size at the transcriptome level while negative at the proteome suggesting inverse RNA-protein dynamic in day 17 SL-II CD16^+^ when compared to PMNs.

Nonetheless, some proteins measured in PMNs lacked corresponding transcripts in both SL-II neutrophils and PMNs. This discrepancy suggests that certain proteins, influencing the proteomic profile of PMNs from blood, may originate from external sources such as plasma or contaminating platelets during the sample preparation for proteomics analysis. To identify these proteins, we selected all proteins in PMNs for which we did not observe matched RNA in either PMNs or CD34^+^ HSPC-derived neutrophils cultured in serum-free SL-II medium at any given time. Using the 136 proteins without an RNA match, we constructed a protein-protein interaction network for known or suspected associations (Figure S3; Table S4). From these, 57 proteins were enriched for plasma/platelet related processes accounting for 42% of the complete protein set. Although PMNs purified from blood were meticulously washed several times during sample preparation, the absence of transcripts for *FGB*, *GP1BB*, *PF4*, *FGG* and *FGA* in serum-free cultured neutrophils indicates that a small fraction of the proteome is enriched for exogenous proteins and clearly do not originate from PMNs themselves.

### CD34^+^ hematopoietic stem and progenitor cell-derived neutrophils gain neutrophil characteristics over time

**Figure 2:**
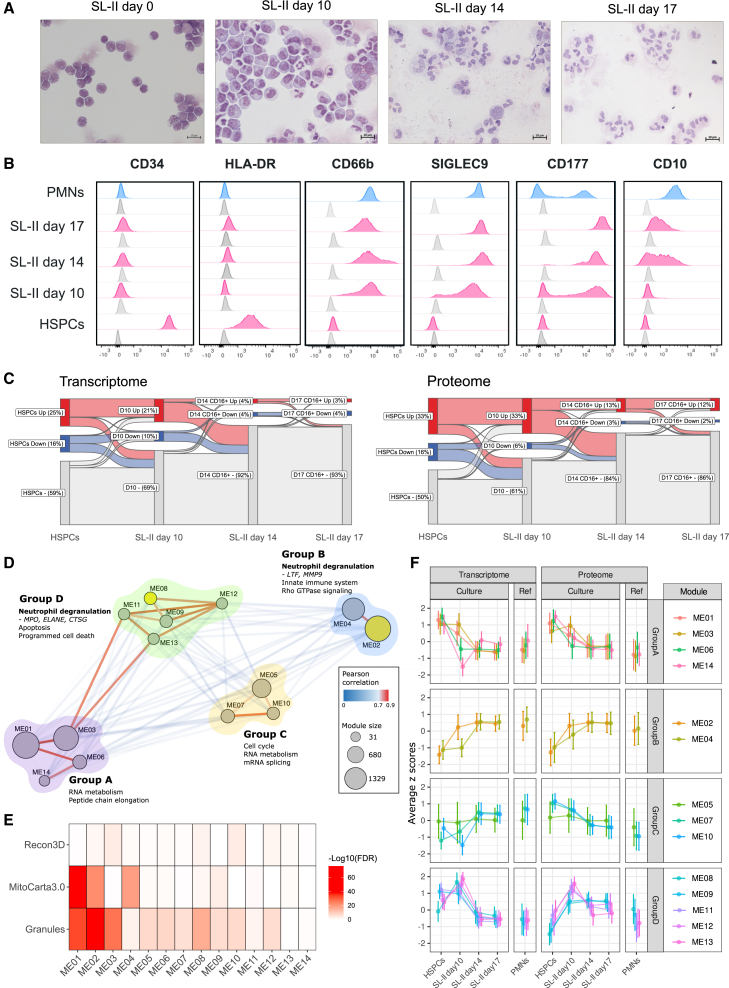
CD34^+^ HSPC-derived neutrophils gain neutrophil characteristics over time (A) Representative cytospins of CD34^+^ HSPC-derived neutrophils cultured in SL-II medium (Bulk) during differentiation on day 0, 10, 14, and 17. Cells were stained with Giemsa-May staining and scalebars were set at 10 μm (*n* = 14). (B) Representative flow cytometric analysis of surface markers CD34, HLA-DR, CD66b, SIGLEC9, CD177, and CD10 expressed on CD34^+^ HSPC-derived neutrophils cultured in SL-II medium (pink) on days 0, 10, and the most mature (CD11b/CD16^+^) fraction on days 14 and 17 compared to PMNs (blue). Histograms of unstained controls are shown in gray (*n* = 6 for CD34, *n* = 4 for HLA-DR, *n* = 8 for CD66b, SIGLEC9, CD177, and CD10). (C) Sankey plot shows the relative proportion of transcripts and proteins that were different when comparing HSPCs on day 0, SL-II Bulk day 10, CD16^+^ SL-II day 14, and CD16^+^ SL-II day 17 to PMNs. Transcripts and proteins that were upregulated are shown in red, downregulated in blue, and non-differentially expressed are shown in gray (FDR <0.05 and log2 fold change| > 1 for proteome and 2 for transcriptome). (D) WGCNA network constructed using z-scores of transcripts and proteins from HSPCs on day 0, SL-II Bulk day 10, CD16^+^ SL-II day 14, and CD16^+^ SL-II day 17 samples. The 14 modules were clustered based on Pearson’s correlation into four different groups (group A, B, C, and D). Blue edges represent Pearson correlation <0.7, and red edges indicate Pearson’s correlations >0.7. Modules that were enriched for neutrophil degranulation are highlighted in yellow. (E) Enrichment heatmap for granule, mitochondrial, and metabolic databases per module. FDR values were obtained from a chi-square test between transcript/protein pairs in modules and each database separately. (F) Scatterplot of the z-scores for transcriptome and proteome along the four stages of differentiation of SL-II in contrast to PMNs for all modules collapsed to the four groups (A–D). Data in (F) is represented as mean ± SD for all transcript/protein pairs included in the modules for PMNs and each stage of SL-II neutrophil differentiation.

To track the differentiation of SL-II CD16^+^ neutrophils, we assessed the myeloid development toward their characteristic neutrophil morphology and immunophenotype on different days. Our cultures contained a low number of cells with a typical neutrophil multi-lobular nucleus at day 10, showing mostly metamyelocytes and band-formed nuclei associated with immature neutrophils (Figure 2A). On day 14 and 17, the majority of cells gained the multi-lobular nuclear morphology, which is characteristic of mature neutrophils. Accordingly, many neutrophil surface markers became increasingly expressed during culturing, as exemplified by CD10, CD66b, and SIGLEC9 (Figure 2B; Table S1). Loss of the HSPC marker CD34 and HLA-DR expression was already observed before day 10 and remained absent in SL-II. In contrast, IMDM cultured cells showed the renewed expression of HLA-DR following day 10 (Figure S2). CD177 showed a bimodal distribution of expression, which relates to epigenetic regulation.^30^^,^^31^ Although heavily skewed toward proportionally more CD177^+^ neutrophils, we did observe a bimodal distribution of CD177 in the cultured neutrophils during earlier stages of the differentiation process, which is in agreement with previous studies on G-CSF-enhanced CD177 expression.^31^^,^^32^^,^^33^

To investigate the myeloid programming of CD34^+^ HSPCs cultured in SL-II medium toward neutrophils at a molecular level, we assessed the transcriptome and proteome of samples collected at different stages of maturation for SL-II culture (i.e., day 0, day 10, day 14 and day 17 of culture). In total, 2,324 genes and 4,405 proteins were different in at least one of these subsets when compared to the starting material (Figure S4). Overall, protein abundances of oxidative phosphorylation and mitochondrial mechanisms decreased while chemokine signaling, motility and phagocytosis related transcripts and/or proteins increased along differentiation. The major deviations for RNA and proteins using PMNs as reference were related to metabolic processes. Cholesterol, glucose, and glycogen metabolism displayed a gradual decrease in transcript abundance from CD34^+^ HSPCs to day 17 SL-II CD16^+^ fraction and seemingly peaking at day 10. However, these abundances at the SL-II day 17 CD16^+^ fraction did not match PMNs. Moreover, we observed a gradual decrease in the number of up- and down-regulated genes along the differentiation of SL-II samples when compared to PMNs (Figure 2C). While this decrease is consistent between transcriptome and proteome, SL-II day 17 CD16^+^ neutrophils have a higher relative proportion of upregulated proteins (12%) in contrast to the transcriptome (3%) and only limited differences were observed between day 14 and day 17 CD16^+^ neutrophils.

Furthermore, to investigate the relationship between transcript and protein abundances along SL-II derived neutrophil differentiation, we implemented a weighted gene co-expression network analysis (WGCNA^34^) using 6,860 transcript/protein pairs for day 0, 10, 14, and 17 together. In total, 14 different modules (ME) were obtained, containing between 31 and 1,329 transcript/protein pairs which were clustered into four groups with two to five MEs per group (Figure 2D; Table S5). To elucidate the biological processes associated with these MEs, a gene over-representation analysis was performed using wiki-pathways^27^ and reactome.^28^ Functional enrichment revealed that MEs in both group A (i.e., ME01, ME03, ME06, and ME14) and group C (i.e., ME05, ME07, and ME10) were associated with essential cellular processes such as RNA metabolism, cell cycle, mRNA splicing, and peptide chain elongation. Groups B (i.e., ME02 and ME04) and D (i.e., ME08, ME09, ME11, ME12, and ME13) were more closely related to neutrophil specific functions such as Rho GTPase signaling and programmed cell death. While both these groups contained MEs with transcript/protein pairs related to neutrophil degranulation (i.e., ME02 and ME08), we observed a clear distinction between specific granule components. The annotation of each module using curated literature-based databases (MitoCarta3.0,^35^ Recon3D^36^ and neutrophil granule components^37^) showed that ME01, ME02, ME04, ME08 and ME09 were associated with mitochondrial activity and ME02, ME03, ME08, ME10 and ME12 associated with metabolic processes (Figure 2E; Table S6). Similarly, except for ME13 and ME14, all other modules were enriched for neutrophil granule components. LTF and MMP9 are members of ME02 and associate with specific and gelatinase granules, while ME08 contained MPO, ELANE, and CTSG, which are azurophilic granule proteins, all showing a clear difference in transcript/protein dynamics along granule development during culture. In particular, MPO and LTF, both with a high connectivity degree ranked by module membership in each respective ME, showed differences in transcript timing, most particularly between day 10 and day 14 (Figure S4).

Furthermore, these MEs displayed diverse global patterns of relative RNA and protein abundance (Figure 2F). When considering the abundance of transcript/proteins together, nine MEs were linearly associated with the time/stage of differentiation (Figure S4). For instance, ME02, ME04 and ME08 showed a positive estimate of association with the time of the maturation of the SL-II medium. In contrast, the effect of transcript/proteins taken together for ME01, ME03, ME06 and ME14 were negatively associated with days of culturing, meaning a decrease in transcript/protein values as differentiation progresses. These associations were not related to the overrepresentation of low abundance transcript/proteins (Figure S4). As for ME08, RNA expression peaked at day 10 and decreased afterward while protein abundance increased along with differentiation. In contrast, ME02 showed an increase of both RNA and protein abundance at day 10, which remained mostly unchanged, mimicking the order of granule formation, i.e., azurophilic granules first, followed by specific and tertiary granules in the bone marrow.^4^ Taken together, changes occurring throughout the culturing of SL-II CD16^+^ neutrophils indicate that these cells lose proliferative capacity, lose their mitochondrial activity, and gain granules while maintaining the capacity to metabolize sugar and fatty acids. These dynamics reflected known stages of differentiation, which relate to the transition of metamyelocytes toward mature neutrophils.^4^

### CD16^+^ cultured neutrophils show a similar microbial killing capacity to blood neutrophils

**Figure 3:**
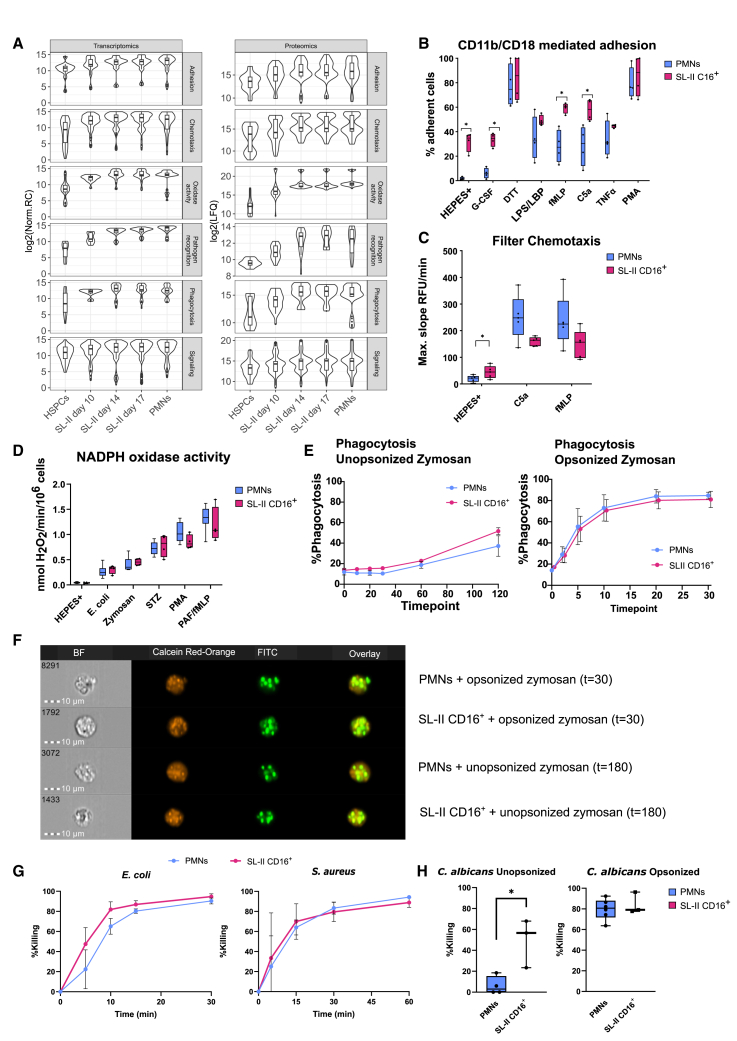
CD16^+^ cultured neutrophils were similar to PMNs in mobility, ROS production, phagocytosis, and microbial killing (A) Boxplots and violin plots showing the relative expression of genes and proteins involved in neutrophil effector functions (Table S7). (B) CD11b/CD18-mediated adhesion assay of PMNs (blue) and SL-II CD16^+^ neutrophils (pink) to uncoated plastic plates (*n* = 4). (C) Chemotactic potential of fluorescently labeled PMNs (blue) and SL-II CD16^+^ neutrophils (pink) based on movement through filters with a pore size of 3 micron (*n* = 5). (D) NADPH oxidase assay to determine the production of extracellular peroxide after the addition of opsonized *E. coli*, zymosan, STZ, PMA, and PAF/fMLP of PMNs (blue) and SL-II CD16^+^ neutrophils (pink) (*n* = 4 for SL-II CD16^+^ neutrophils and *n* = 8 for PMNs). (E) Phagocytosis of either unopsonized or opsonized zymosan by PMNs (blue) versus SL-II CD16^+^ neutrophils (pink) measured by flow cytometry (*n* = 3 for SL-II CD16^+^ neutrophils and *n* = 4 for PMNs). (F) Representative images of phagocytosis of either unopsonized or opsonized zymosan labeled with FITC solution (green) by PMNs labeled with calcein red-orange (orange) versus SL-II CD16^+^ neutrophils labeled with calcein red-orange (orange) at the latest timepoint as visualized by imaging flow cytometric analysis. Scale bar was set at 10 μm (*n* = 3). (G and H) Killing of opsonized *E. coli* and *S. aureus,* and unopsonized and opsonized *C. albicans,* respectively, shown for PMNs (blue) (*n* = 3) versus SL-II CD16^+^ neutrophils (pink) (*n* = 3). Killing was quantified as the inverse of colony-forming units (CFU) as a percentage relative to the CFU at the start of the assay. Negative values, signifying an increase in the number of colonies were considered to be 0. Data shown in (A–D, H) is represented as median and interquartile range, and (E) and (G) is represented as mean ± SD. *p* values were calculated using Mann-Whitney U tests and labeled as ∗*p* < 0.05 and ∗∗*p* < 0.01. *n* values represent the number of individual donor samples.

Having established the similarities and differences of cultured neutrophils compared to PMNs on a molecular level and their overall differentiation dynamics from an unbiased approach, we next compiled a list of proteins important for adhesion, chemotaxis, oxidative activity and phagocytosis, as well as intracellular signaling (Table S7). Neutrophil-specific processes such as phagocytosis, oxidase activity, and pathogen recognition displayed a gradual increase in RNA expression followed by increased protein abundance, with day 17 SL-II CD16^+^ most closely resembling PMNs (Figure 3A). No substantial shifts in the distribution of transcript and protein abundance were observed along differentiation for genes involved in intracellular signaling (i.e., Rho GTPase). Next, we investigated how this translated to the functionality of day 17 SL-II CD16^+^ cells and compared that to PMNs. Both MACS-purified SL-II CD16^+^ cells and PMNs showed an increase in CD11b/CD18-mediated adhesion upon stimulation (Figure 3B). However, SL-II CD16^+^ neutrophils adhered to a polystyrene plate without stimulation (HEPES+), which can be ascribed to the G-CSF stimulation while being cultured.^38^ SL-II Bulk cells showed a lower adhesive background (Figure S5), in large part due to the presence of more immature cells, which do not yet adhere to the plate. Furthermore, Bulk samples from SL-II and IMDM behaved similarly in the adhesion assay, emphasizing the importance of sorting for the most mature cells in the cultures to properly study their functional capacity.

Furthermore, we assessed the chemotactic ability of SL-II CD16^+^ neutrophils (Figure 3C). Cultured SL-II CD16^+^ neutrophils responded to both fMLP and C5a, as did PMNs. They showed slower movement when compared to PMNs, albeit not statistically significant. The cultured SL-II CD16^+^ neutrophils showed a higher spontaneous motility (i.e., chemokinesis), as is associated with G-CSF priming.^38^^,^^39^ Endpoint measurements showed no differences in fluorescence (Figure S5), which implies that, although slower, a similar number of cells passed through the filter. When assessing chemotaxis in SL-II and IMDM cultures in Bulk, a significantly lower response is observed compared to the most mature fraction of SL-II CD16^+^ neutrophils (Figure S5), indicating that neutrophil motility develops upon full maturation. Endpoint measurements showed a lower response for Bulk SL-II and IMDM compared to PMNs (Figure S5). To further dissect the slight difference in the chemotactic ability of cultured cells with PMNs, we assessed chemotaxis with the Taxiscan assay (Videos S1, S2, S3, and S4). Based on movement on uncoated glass, SL-II CD16^+^ neutrophils showed directionality and responded to stimuli, although moving slightly slower compared to PMNs after stimulation. Thus, our cultured neutrophils seem fully capable of chemotactic movement.

Video S1. Representative video of the taxiscan assay showing the unstimulated chemotactic capability of PMNs (*n* = 4) HEPES+ medium was used as a negative control. N values represent the number of individual donor samples.

Video S2. Representative video of the taxiscan assay showing the unstimulated chemotactic capability of SL-II CD16^+^ cultured neutrophils (*n* = 4) HEPES+ medium was used as a negative control. N values represent the number of individual donor samples.

Video S3. Representative video of the taxiscan assay showing the chemotactic capability of PMNs toward fMLP used as chemoattractant (*n* = 4) N values represent the number of individual donor samples.

Video S4. Representative video of the taxiscan assay showing the chemotactic capability of SL-II CD16^+^ cultured neutrophils toward fMLP used as chemoattractant (*n* = 3) N values represent the number of individual donor samples.

Several mechanisms are known to contribute to neutrophil killing activity, including ROS production and pathogen phagocytosis. To this end, we compared cultured neutrophils to PMNs in terms of NADPH oxidase dependent ROS production (Figure 3D). SL-II CD16^+^ neutrophils showed similar production of ROS for all tested stimuli. In contrast, the Bulk fraction of both SL-II and IMDM cultures showed decreased ROS production compared to PMNs (Figure S5). Additionally, uptake of unopsonized and opsonized FITC-labelled zymosan by SL-II CD16^+^ neutrophils was comparable to that of PMNs as analyzed by flow cytometry and visualized by imaging flow cytometry data (Figures 3E and 3F). When incubated with microbial pathogens, both SL-II CD16^+^ neutrophils and PMNs were able to kill opsonized *E. coli*, opsonized *S. aureus,* and opsonized *C. albicans* equally, whereas SL-II CD16^+^ neutrophils seemed even better equipped to kill unopsonized *C. albicans* (Figures 3G and 3H). Taken together, SL-II CD16^+^ neutrophils resemble PMNs in terms of movement and are fully capable of pathogen killing.

### CD16^+^ cultured neutrophils show high antibody-dependent cellular cytotoxicity but are deficient in T cell suppression

**Figure 4:**
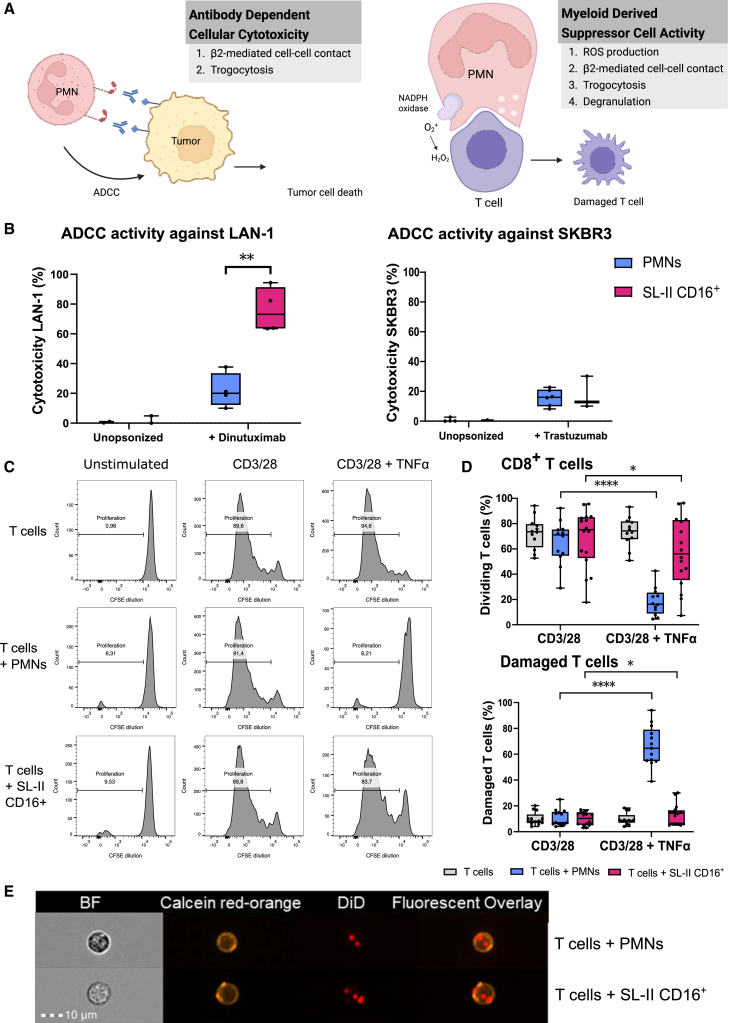
SL-II CD16^+^ neutrophils retain tumor cell killing capacity but are deficient in immunosuppressive capacity (A) Cartoon of antibody dependent cellular cytotoxicity (ADCC) and myeloid derived suppressor cell (MDSC) activity exerted by mature activated neutrophils (Figure adapted from Aarts et al.^14^). (B) *In vitro* ADCC of LAN-1 cells unopsonized and opsonized (+Dinutuximab) (*n* = 4) and SKBR3 cells unopsonized and opsonized (+Trastuzumab) by PMNs (blue) (*n* = 6) and SL-II CD16^+^ neutrophils (pink) (*n* = 3) in a 1:50 T:E ratio. (C) Representative CFSE plots for CD8^+^ T cell proliferation of an *in vitro* MDSC activity assay. T cells were stimulated with anti-CD3/CD28 antibodies to induce proliferation and co-cultured with either unstimulated or TNFα-stimulated PMNs or SL-II CD16^+^ neutrophils. After 4 days, T cell proliferation was assessed by CFSE dilution (*n* = 12 for T cells alone and *n* = 16 for co-culturing with PMNs or SL-II CD16^+^ neutrophils). (D) Boxplots of CD8^+^ T cell proliferation and damaged T cell formation in the *in vitro* MDSC assay. Unstimulated or TNFα stimulated PMNs (blue) (*n* = 12) or SL-II CD16^+^ neutrophils (pink) (*n* = 16) were co-cultured with T cells. After 4 days, FSC/SSC gating was used to assess ‘damaged’ T cell formation next to T cell proliferation. (E) Representative images taken with imaging flow cytometry of trogocytosis carried out by PMNs and SL-II CD16^+^ neutrophils. The scale bar was set at 10 μm (*n* = 3). PMNs and SL-II CD16^+^ neutrophils were stained with calcein red-orange (orange), and T cells were stained with DiD (red) before co-culturing for 4 h. Data in (B) and (D) is represented as median and interquartile range. *p* values were calculated using Mann-Whitney U tests and labeled as ∗*p* < 0.05, ∗∗*p* < 0.01, and ∗∗∗∗*p* < 0.0001. *n* values represent the number of individual donor samples.

Knowing that the basic neutrophil functions of SL-II CD16^+^ neutrophils resemble PMNs, we further investigated neutrophil activity in the context of tumor or other immune cells (Figure 4A). Our flow cytometric analysis showed that SL-II CD16^+^ neutrophils express FcyRII (CD32; Table S1), thus we assayed their ADCC capacity. ADCC activity by SL-II CD16^+^ neutrophils was enhanced for the neuroblastoma cell line LAN-1 in comparison to the activity exerted by PMNs (Figure 4B), which could be explained by the presence of G-CSF in the culture medium working as an activator for ADCC activity.^40^ When comparing SL-II CD16^+^ neutrophils to GM-CSF-stimulated blood neutrophils, we observe a higher killing capacity for PMNs (Figure S6). ADCC activity for the Her2Neu-positive breast cancer cell line SKBR3 was lower but comparable between SL-II CD16^+^ neutrophils and PMNs. Stimulation with GM-CSF increased the killing capacity of PMNs (Figure S6).

Neutrophils can also exert immunosuppressive activity, known as MDSC activity, which requires ROS production, β2-mediated cell-cell contact, trogocytosis, and degranulation. As previously shown, PMNs only perform MDSC activity upon *in vitro* stimulation to irreversibly suppress T cell activation, including proliferation and cytokine production, leading to damaged T cells, which can be assessed using flow cytometry to gate on formerly called ‘small T cells’^14^ (Figure S6). Upon TNFα stimulation, PMNs strongly inhibited CD8^+^ T cell proliferation, while this inhibition was reduced when T cells were co-cultured with SL-II CD16^+^ neutrophils (Figures 4C and 4D). Similar observations were found for PMNs and SL-II CD16^+^ co-cultured with CD4^+^ T cells (Figure S6). Moreover, co-culturing T cells with TNFα-stimulated PMNs showed an increased population of damaged T cells mirroring the suppressive effects on T cell proliferation, while SL-II CD16^+^ neutrophils did not show the formation of damaged T cells to the same extent in these standard MDSC activity assays.

Since MDSC activity in SL-II CD16^+^ neutrophils was abrogated, and we demonstrated that β2-mediated adhesion and neutrophil NADPH oxidase activity were similar for SL-II CD16^+^ neutrophils as for PMNs, we assessed the ability of SL-II CD16^+^ neutrophils to perform trogocytosis by imaging flow cytometry. Representative images show that both PMNs and SL-II CD16^+^ neutrophils are capable of taking up multiple pieces of T cell membrane (Figure 4D). To formally prove the internalization of T cell membrane, we performed confocal 3D Live Cell Imaging (Zeiss LSM 980 Airyscan 2) using a similar setup, which confirmed that these small pieces of T cell membrane were indeed inside the SL-II CD16^+^ neutrophils (Video S5). Overall, these data show a deficiency in T cell suppression for SL-II CD16^+^ neutrophils, while β2-mediated cell-cell contact, ROS production, and trogocytosis were intact (Figure 4D).

Video S5. Representative video of SL-II CD16^+^ cultured neutrophils performing trogocytosis of T cells (*n* = 5) Here, we display a neutrophil containing a piece of T cell membrane inside the cytoplasm. The SL-II CD16^+^ cultured neutrophil is shown in orange, and the T cell membrane is shown in pink. N values represent the number of individual donor samples.

We subsequently investigated the expression of transcripts and proteins described as a possible MDSC signature in previous studies,^41^^,^^42^ being mostly based on mouse studies and single-cell sequencing approaches of intra-tumor neutrophils in humans. For instance, transcripts of ARG1, CD52, CD84, FATP2, FFAR2, LOX-1, and PTGER2 had previously been suggested to be important to identify the immunosuppressive neutrophils with MDSC activity.^42^^,^^43^^,^^44^^,^^45^ From these, transcript abundance was increased for FATP2 and decreased for FFAR2 in SL-II CD16^+^ neutrophils compared to PMNS (FDR <0.05; |Log2LFC| > 2). Subsequently, only the protein abundance of LOX-1 was significantly lower in SL-II CD16^+^ neutrophils compared to PMNs (FDR <0.05, |Log2LFC| > 1; Table S2). However, the surface marker expression for LOX-1 and CD84 was similar between PMNs and cultured CD16^+^ neutrophils (Figure S6). The discrepancies observed in LOX-1 might be explained by the detection method used, where mass spectrometry measures complete cellular LOX-1 abundance, and flow cytometry is limited to surface expression. In contrast, the surface expression of CD52 was absent on the PMN fraction, and it was detected in cultured neutrophils, whereas it was not detected in the proteome of either fraction. This leaves the observed functional difference in MDSC activity as such largely unexplained by this molecular component alone. Nevertheless, understanding the precise molecular mechanism linking MDSC activity to this molecular signature was beyond the scope of the current study.

### CD16^+^ cultured neutrophils show a distinct granular content compared to polymorphonuclear cells

**Figure 5:**
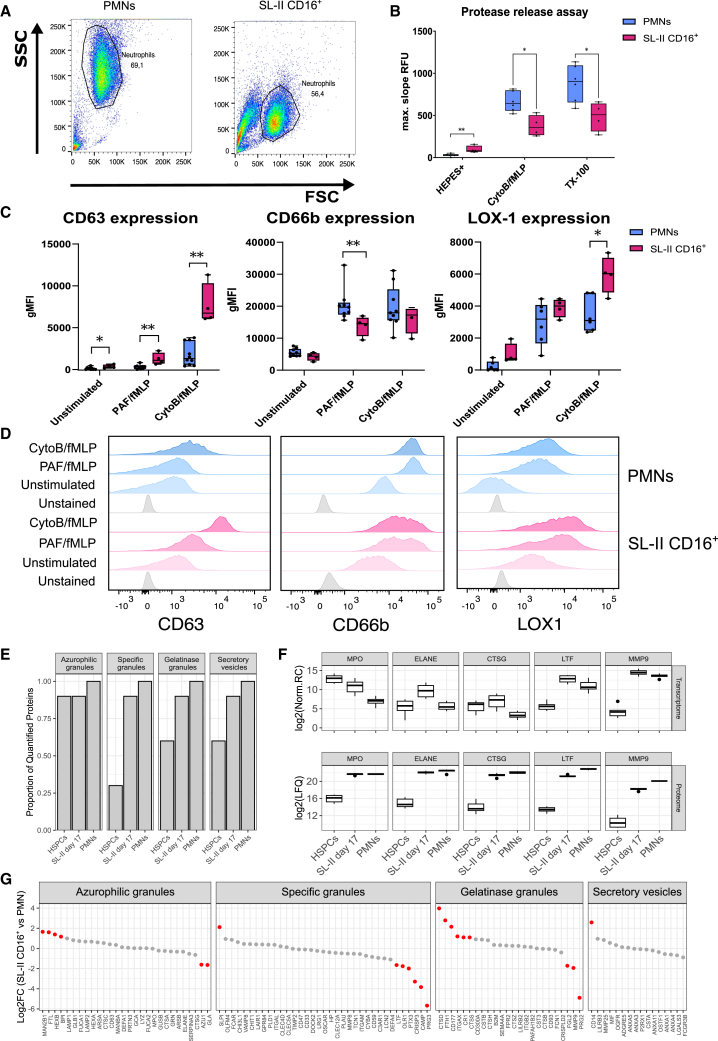
SL-II CD16^+^ neutrophils were able to release granules but showed granular content distinct from PMNs (A) Representative FSC/SSC plots measured by flow cytometry for PMNs and SL-II CD16^+^ neutrophils (*n* = 14). (B) Release of serine-proteases ELANE and CTSG as measured by the maximal combined degradation of DQ-BSA after stimulation with CytoB and fMLP. Lysis of cells with Tx-100 was used as a control for maximal release. (*n* = 4). (C) Geometric mean florescence intensity of CD63 (azurophilic granules) (*n* = 10 for PMNs and *n* = 4 for SL-II CD16^+^ neutrophils), CD66b (specific granules) (*n* = 9 for PMNs and *n* = 4 for SL-II CD16^+^ neutrophils) and LOX-1 (specific granules) (*n* = 6 for PMNs and *n* = 4 for SL-II CD16^+^ neutrophils) on PMNs (blue) and SL-II CD16^+^ neutrophils (pink) in unstimulated conditions, after stimulation with PAF/fMLP or CytoB/fMLP measured by flow cytometry. Negative values were considered to be 0. (D) Representative histograms for degranulation (gMFI shown in C) by PMNs (blue) and SL-II CD16^+^ neutrophils (pink) under different conditions. Histograms of unstained controls are shown in gray. (E) Proportion of valid values for granule proteins (Table S8) for CD34^+^ HSPCs, CD16^+^ SL-II neutrophils, and PMNs. (F) Boxplots showing the log2 normalized read counts and log2 LFQ for signature granule proteins (i.e., MPO, ELANE, CTSG, LTF, and MMP9) for CD34^+^ HSPCs, CD16^+^ SL-II neutrophils, and PMNs. (G) Scatterplot of the effect size estimates for granule proteins when comparing CD16^+^ SL-II neutrophils to PMNs. Granule proteins were separated into azurophilic granules, specific granules, gelatinase granules, and secretory vesicles. Dots highlighted in red are considered statistically significant after multiple testing correction (FDR <0.05) and |log2 fold change| > 1. Data in (B), (C), and (F) is represented as median and interquartile range. *p* values were calculated using Mann-Whitney U tests and labeled as ∗*p* < 0.05, ∗∗*p* < 0.01. *n* values represent the number of individual donor samples.

Having no phenotypic marker that would help to clearly indicate a defect in MDSC activity, we now focused on the remaining functional requirements for immunosuppressive activity, i.e., degranulation and granule content, while knowing that the other functional requirements for MDSC activity (i.e., cell-cell contact, trogocytosis and ROS production) were comparable between SL-II CD16^+^ neutrophils and PMNs. FSC and SSC plots from flow cytometric analyses suggested a difference in granularity between SL-II CD16^+^ neutrophils and PMNs (Figure 5A). To confirm a difference in granularity, we assessed the capacity of these neutrophils to release proteases by cleavage of the probe DQ-BSA, which is mediated by the azurophilic granule proteins ELANE and CTSG (Figure 5B). SL-II CD16^+^ neutrophils show reduced proteolytic activity after the stimulation of cytochalasin-preincubated fMLP-induced neutrophils (CytoB/fMLP), when compared to PMNs from blood. However, the total protease content released upon lysis with Triton X-100 was also reduced for SL-II CD16^+^ neutrophils.

We then assayed degranulation by measuring the upregulation of surface markers after the stimulation of neutrophils. This was considered to be normal by the upregulation of surface markers typically associated with the release of azurophilic granules (i.e., CD63) and specific granules (i.e., CD66b, LOX-1) content (Figures 5C and 5D). The expression of these membrane proteins increased after stimulation to levels found on activated PMNs for CD66b and was even higher for CD63 and LOX-1. Subsequently, we assessed differences in granule protein content between HSPCs, SL-II CD16^+^ neutrophils, and PMNs using a curated list of granule proteins described in the literature.^4^^,^^37^ This curated list of 111 granule proteins is shown in Table S8. From these, 80% of the azurophilic granule proteins and about 75% of specific granule proteins were present in both SL-II CD16^+^ neutrophils and PMNs (Figure 5E). As a relative proportion, a similar fraction of proteins associated with azurophilic granules observed in SL-II CD16^+^ and PMNs were already observed in the G-CSF-mobilized CD34^+^ HSPCs at the start, suggesting the proportion of cells with a certain amount of these proteins – even when minute – did not vary along differentiation. In contrast, large differences in the proportion of proteins associated with specific granules, gelatinase granules and secretory vesicles were observed between CD34^+^ HSPCs and either SL-II CD16^+^ or PMNs, where the latter two resemble each other. To identify quantitative differences in granule protein content, we compared SL-II CD16^+^ to PMNs (Figure 5F). SL-II CD16^+^ neutrophils were clearly deficient in specific granule proteins such as CAMP, CRISP3 and PRG3, and including OLR1 (i.e., LOX-1). Similarly, these cells were also deficient in gelatinase granule proteins FGL2 and MMP9, with greatly reduced levels of PRG2. Protein levels of CTSD and FTH1 in gelatinase granules, SLPI in specific granules, and membrane-associated CD14 in secretory vesicles, were higher in SL-II CD16^+^ neutrophils compared to PMNs. Higher transcript abundances for *MPO*, *ELANE*, *CTSG*, *LTF* and *MMP9* were observed in SL-II CD16^+^ cells in contrast to PMNs, while the protein levels of these and other known granular proteins were much lower in HSPCs (Figure 5G). Together, these data suggest a distinct granular protein content for SL-II CD16^+^ neutrophils compared to PMNs, in particular for the specific and tertiary granule components, which could correlate with the deficiency in MDSC activity.

## Discussion

In this study, we developed a serum-free culturing system which leads to neutrophils with segmented nuclei and functional characteristics of a mature effector cell. These neutrophils are much closer to healthy donor blood neutrophils than the cells generated by currently used standard culturing schemes, as most of these protocols for HSPCs rely on the use of IMDM supplemented with growth factors and fetal calf serum (FCS).^21^^,^^23^^,^^24^^,^^46^^,^^47^^,^^48^ Although these protocols are able to produce neutrophils with segmented nuclei, many do not report on the expression of surface markers CD66b and CD16, and functional testing is incomplete.^23^^,^^24^^,^^46^^,^^48^

We extensively characterized both IMDM and SL-II derived neutrophils in phenotype and functionality, showing that end-stage (day 17) SL-II derived neutrophils closely resemble blood neutrophils (CD15^+^CD11b^+^CD16^high^CD66b^+^), whereas IMDM derived neutrophils show a different phenotype (CD15^+^CD11b^+^CD16^low/dim^CD66b^+^CD14^high^CD29^high^). Both transcriptomic and proteomic analysis underscore that end-stage SL-II CD16^+^ neutrophils show high similarity to circulating neutrophils. Our data suggest some metabolic pathways, such as cholesterol, fatty acid and glucose/glycogen remain active and are not yet depleted to the same level as in PMNs. During early stages of hematopoiesis, a shift from glycolysis toward oxidative phosphorylation is observed to most likely meet the energy requirements for proliferation and myeloid differentiation.^49^ Although close in function and phenotype, overall genes and proteins related to metabolic activity, including cell cycling, ribosomal translation, electron transport chain and mitochondrial oxidative phosphorylation decreased over time in culture, but not matching the level observed in PMNs. In fact, there is a difference in the overall maturation state of SL-II CD16^+^ neutrophils compared to PMNs. For instance, SL-II CD16^+^ neutrophils do not match the expected decrease of mitochondrial activity which, at later stages of granulopoiesis, is rapidly reduced in human neutrophils developing in the bone marrow.^4^ This is furthermore emphasized by the reduced abundance of effector proteins, most strongly for those related to specific and gelatinase granules, which originate at later stages of granulopoiesis. Notwithstanding these subtle differences, the final effector functions such as motility and microbial killing, were intact. Taken together, although not reaching full maturation, our cultured cells closely resemble PMNs for host defense functions, and more so compared to the IMDM counterparts.^21^^,^^22^^,^^24^

Human MDSC activity has been mostly investigated using single cell RNAseq approaches to suggest phenotypic signatures for immunosuppressive intratumor behavior.^42^ In the SL-II cultured neutrophils, the transcriptomic signature was largely intact except for the abundance of transcripts of *FATP2* and *FFAR2* and the protein LOX-1, suggesting that these genes might not encompass the complete MDSC phenotype. Instead, the findings in this culture model indicate that the immunosuppressive MDSC activity may also be dependent on a final stage of maturation reaching similar levels of specific and gelatinase granule proteins when compared to PMNs. However, this may not occur under the G-CSF culture conditions used in this study. These results indicate that caution should be exercised when interpreting single-cell RNA signatures for MDSC activity. For this reason, to evaluate MDSC activity of SL-II cultured neutrophils, we took a functional approach as a gold standard. Many of the mechanistic processes determined to be involved in MDSC activity of human neutrophils were intact including adhesion, ROS formation, MPO abundance and release, as well as trogocytosis.^14^ A close comparison of SL-II CD16^+^ neutrophils and PMNs indicated a difference in granule content. The azurophilic granule content is very similar, degranulation was intact, which is sufficient for microbial killing, whereas specific and gelatinase granules, developing during later stages in these cultures, lack some of their protein content. The deficiency in granular content in SL-II CD16^+^ neutrophils is not as absolute as in patients with specific granule deficiencies^50^ and is unequally distributed over the various proteins stored in the specific and gelatinase granules of PMNs. At the protein level, ARG1, previously reported to be involved in MDSC activity,^51^ showed no difference, while a reduced protein abundance of CAMP, CRISP3, PRG2, and PRG3 was observed in the cultured neutrophils, which have not been associated with any immunosuppressive characteristic of neutrophils before. Some of the specific granule components contribute to the immunosuppressive activity of PMNs, which would support data from previous work in patients with a partial or complete defect in specific granules, known as the genetically NBEAL2-deficient Gray Platelet Syndrome and Specific Granule Disorder, respectively, which also lack MDSC activity^50^ (unpublished data). Although it is unclear whether, and to what extent, any of these components may be critically involved in the immunosuppressive activity of neutrophils, the differences in T cell suppression observed in SL-II CD16^+^ neutrophils may be associated with the lack of sufficient MDSC activity by their overall granular content.

Over the past decade, the importance of neutrophil research has increased, in particular for cancer biology^52^^,^^53^^,^^54^ in part by the use of their ADCC capacity.^55^^,^^56^^,^^57^^,^^58^^,^^59^^,^^60^ In this study, we have shown that SL-II CD16^+^ neutrophils are capable of killing opsonized LAN1 cells without requiring activation by GM-CSF, which is required for efficient killing by PMNs. The addition of G-CSF in the culture medium during differentiation might provide the activation necessary for unleashing cytotoxic potential.^40^ Creating a neutrophil transfusion product with a potentially strong anti-tumor signature without immunosuppressive activity could become an option, especially against tumors that other immune cells find notoriously difficult to infiltrate.^25^ Challenges include the limited amount of source material, as CD34^+^ HSPCs are isolated from either cord blood or mobilized blood. Granulocyte transfusions range from 0.6 to 1.0·10^9^ granulocytes per kilogram bodyweight, meaning that 60 to 80 kg individuals need to receive 36–80·10^9^ granulocytes per transfusion.^61^^,^^62^^,^^63^^,^^64^ In order to culture these numbers with the current protocol would require at least 360·10^6^ HSPCs, as we measured a 100 to 200-fold expansion of HPSCs to neutrophils (360·10^6^ multiplied by 100 results in 36·10^9^ cultured neutrophils). As one donation of mobilized peripheral blood at 500 mL would contain approximately 0.5–1.0·10^9^ HSPCs, this process would be heavily dependent on donors. This could be remedied either by the potent *ex vivo* expansion of CD34^+^ HSPCs^65^ or *de novo* generation of iPSC-derived CD34^+^ HSC-like cells.^66^

Our data also indicate the potential plasticity of human neutrophils to obtain functions that may otherwise only exist under certain conditions.^67^ Although reliant on serum and inferior to SL-II CD16^+^ neutrophils in terms of motility, about 30% of IMDM cultured neutrophils hold a unique feature due to their expression of HLA-DR. Previous work has shown that human neutrophils in a tumor environment can gain HLA-DR expression, which correlates with better prognosis for several cancer types.^68^ Also, human neutrophils treated with leucine or arginine *in vitro*, as well as neutrophils from mice on a leucine rich diet could express HLA-DR.^68^ This plasticity of neutrophils may depend on exposure to circulating factors within the bone marrow niche during their development,^68^ and it can be induced in PMNs.^69^ Nevertheless, whether HLA-DR^+^ neutrophils could potentially be useful for clinical application may be further studied.

In this study, we have performed an extensive analysis of neutrophil cultures that can be used as a research model, allowing the study of genetic modifications. In addition, we provided clues to the incompletely understood mechanisms of immunosuppression by neutrophils. Through the distinct differences between SL-II CD16^+^ neutrophils and those from blood, our data suggest that granule development might be important for the immunosuppressive capacity of blood neutrophils toward T cells. Additionally, although several challenges still remain, this culture method holds the potential to become a distinct cell therapy in its own right.

### Limitations of the study

In this study, we showed that neutrophils derived from CD34^+^ HSPCs cultured in serum free medium exhibit phenotypic, molecular, and functional similarities to PMNs. However, both HSPCs and PMNs were obtained from a relatively small pool of donors, with an even sex distribution but a large age range (16–66 years). As a result, we were unable to assess the potential effect of donor characteristics on the observed outcomes. Furthermore, due to the use of Bulk samples, our dissection of molecular mechanisms in the SL-II culture neutrophils is limited. This is evident by the presence of eosinophil, plasma, and platelet transcript/protein pairs, which might be attributed to impure fractions. Nonetheless, the presence of contaminants has been shown in previous studies using similar approaches, where eosinophil-specific transcripts and proteins have been detected in fractions depleted for eosinophils.^4^ The identification of these proteins can also be attributed to the increased resolution of current MS-MS techniques. Similar issues have been encountered when measuring purified plasma samples.^70^ Nonetheless, we have tried to identify exogenous plasma and platelet proteins by successfully integrating transcript and protein presence between serum-free SL-II cultured neutrophils and vigorously washed PMNs from blood. Furthermore, we acknowledge that single-cell sequencing or proteomics approaches would improve the identification of the heterogeneity among myeloid cells going through the different stages of maturation while attenuating the effect of exogenous sources in PMN and cultured fractions. Nonetheless, we aimed for an integrated, detailed analysis of the omics approaches used, for which single-cell proteomics does not yet reach the desired depth to do so. Further studies might achieve a higher precision in elucidating the molecular mechanisms behind the stages of culture maturation, making use of single-cell techniques.

## Resource availability

### Lead contact

Requests for further information and resources should be directed to and will be fulfilled by the lead contact, Prof. Taco W. Kuijpers, MD PhD (t.w.kuijpers@amsterdamumc.nl).

### Materials availability

This study did not generate new unique reagents.

### Data and code availability

• **Data:** Raw Bulk RNAseq data (FASTQ) files can be accessed via the Gene Expression Omnibus (GEO) portal with accession number GSE300707. Raw and processed proteomics data have been deposited to the ProteomeXchange Consortium via the PRIDE^71^ partner repository with dataset identifier: PXD060579. All data is publicly available as of the date of publication and is referred to in the key resources table.
• **Code:** All original code used for transcriptomics, proteomics, and WGCNA analysis has been deposited at GitHub: www.github.com/FelipeRojasR/Neutrophils_SLII.
• **Additional information:** Any additional information and data related to this article are available from the lead contact upon request.

## Acknowledgments

We wish to thank all donors who have contributed blood to this study. We wish to thank the people from the Sanquin Central Facility, especially Erik Mul and Simon Tol, for helping us with setting up the imaging flow cytometry experiments. We also thank our lab technicians, Anton Tool, Paul Verkuijlen, and Karin Schornagel, for helping us with lab work and participating in work discussions. We would like to thank Céline Mahieu for the critical reading of the article. We would like to thank Erfan Nur for his generous gift of G-CSF. This work was supported by the Dutch Ministry of Health (PPOD 2313, PPOC 1922, PPOC 2089), E-Rare-3 JTC2018SF (LADOMICS) and Stichting Universitas.

## Author contributions

S.D.S.W., A.A.F.G., F.v.O., D.S.K., A.K.K., A.J.H., and J.G. performed experiments and collected data. F.R.R. and A.J.H. performed computational and statistical modeling. S.D.S.W., A.A.F.G., F.v.O., F.R.R., H.T., A.J.H., and T.W.K. contributed to the design of the study. S.D.S.W., A.A.F.G., F.v.O., F.R.R., D.S.K., A.A.K., R.v.B., H.L.M., H.T., A.J.H., and T.W.K. evaluated data and interpreted biological relevance. S.D.S.W., A.A.F.G., F.v.O., F.R.R., A.J.H., H.T., and T.W.K. wrote the initial draft of the article. A.J.H., T.J.P.v.D., H.T., and T.W.K. were involved in supervision. All authors approved the article.

## Declaration of interests

The authors declare no competing interests.

## STAR★Methods

### Key resources table

**Table:** 

REAGENT or RESOURCE	SOURCE	IDENTIFIER
**Antibodies**		

APC mouse anti-human CD4 (Clone SK3)	BD Biosciences	Cat #566915; RRID:AB_2739445
PerCP/Cyanine5.5 anti-human CD8 Antibody (Clone SK1)	Biolegend	Cat #344709; RRID:AB_2044009
PE Mouse Anti-Human CD10 (Clone HI10α)	BD Biosciences	Cat #555375; RRID:AB_395776
Pacific Blue™ Mouse Anti-Human CD11b (Clone ICRF44)	BD Biosciences	Cat #558123; RRID:AB_397043
CD11b APC (Clone D12)	BD Biosciences	Cat #333143; RRID:AB_2868644
PE-Cy™7 Mouse Anti-Human CD14 (Clone M5E2)	BD Biosciences	Cat #557742; RRID:AB_396848
CD15 FITC (Clone MMA)	BD Biosciences	Cat#332778; RRID:AB_2868627
CD15 Monoclonal Antibody (Clone HI98), PE	Thermofisher Scientific	Cat #12-0159-42; RRID:AB_11219674
Pacific Blue Mouse Anti-Human CD16 (3G8)	BD Biosciences	Cat #558122; RRID:AB_397042
PE-Cy™7 Mouse Anti-Human CD16 (Clone 3G8)	BD Biosciences	Cat #557744; RRID:AB_396850
PE Mouse Anti-Human CD29 (Clone MAR4)	BD Biosciences	Cat #5554433; RRID:AB_395836
Mouse anti Human CD32 (Clone AT10)	Bio-Rad	Cat #MCA1075F; RRID:AB_321660
PE Mouse Anti-Human CD33 (Clone WM53)	BD Biosciences	Cat #555450; RRID:AB_395843
APC anti-human CD34 Antibody (Clone 581)	BioLegend	Cat #343510; RRID:AB_1877153
BD™ CD36 FITC (Clone CLB-IVC7 (ASR))	BD Biosciences	Cat #656152; RRID:AB_2870401
APC Anti-Human CD41 (Clone HIP8)	Biolegend	Cat #984504; RRID:AB_2941650
APC Mouse Anti-Human CD45 (Clone HI30)	BD Biosciences	Cat #555485; RRID:AB_398600
FITC anti-human CD52 Antibody	BioLegend	Cat #316004; RRID:AB_389277
BD™ CD61 FITC (Clone RUU-PL7F12 (CE_IVD))	BD Biosciences	Cat #347407; RRID:AB_2868851
APC Mouse Anti-Human CD62L (DREG-56)	BD Biosciences	Cat #559772; RRID:AB_398668
Human anti CD63 (Clone MX-49/129.5) Alexa Fluor 647	Santa Cruz Biotechnology	Cat #sc-5275 AF547; RRID:AB_627877
Mouse anti Human CD64 antibody (Clone 10.1)	Bio-Rad	Cat #MCA756F; RRID:AB_321799
Mouse Anti-Human CD66b Monoclonal antibody, FITC Conjugated (Clone 80H3)	Bio-Rad	Cat #MCA216F; RRID:AB_2077860
CD69 Monoclonal Antibody (Clone FN50), PE-Cyanine7	Thermofisher Scientific	Cat #25-0699-42; RRID:AB_1548714
Human anti CD84	BioLegend	Cat #326002; RRID:AB_830813
Goat anti-Mouse IgG (H+L), Cross-Adsorbed Secondary Antibody, Alexa Fluor ™ 488	ThermoFisher Scientific	Cat #A11001; RRID:AB_2534069
PE anti-human CD88 (C5aR) Antibody (Clone S5/1)	BioLegend	Cat #344304; RRID:AB_2067175
PerCP/Cyanine5.5 anti-human CD101 (Clone BB27) Antibody	BioLegend	Cat #331016; RRID:AB_2716110
Human anti CD177	Bio-Rad	Cat #MCA2045F; RRID:AB_323349
FITC Annexin V	BD Biosciences	Cat #556419; RRID:AB_2665412
Hamster anti human EMR3:FITC (Clone 3D7)	Bio-Rad	Cat #MCA2476F; RRID:AB_872002
Human FPR1 Fluorescein-conjugated Antibody (Clone 350418)	R&D Systems	Cat #FAB3744F; RRID:AB_2105317
FITC Mouse Anti-Human HLA-DR (Clone G46-6)	BD Biosciences	Cat #555811; RRID:AB_396145
PE anti-human LOX-1 Antibody (Clone 15C4)	BioLegend	Cat #358603; RRID:AB_2562180
PE anti-human CD274 (B7-H1, PD-L1) Antibody (Clone 29E.2A3)	BioLegend	Cat #329706; RRID:AB_940368
PE anti-human Siglec-9 Antibody (Clone K8)	BioLegend	Cat#351503; RRID:AB_10722615

**Bacterial and virus strains**		

*Escherichia coli*		Strain ML35
*Staphylococcus aureus*		Strain 502A

**Chemicals, peptides, and recombinant proteins**		

C5a	Sino Biological Inc	Cat#10604-HNAE
Calcein-AM	Molecular Probes	Cat#C3100MP
Calcein red-orange	ThermoFisher Scientific	Cat#C34851
CD3/CD28 Anti-CD3 (Clone 1XE [IgE isotype]) Anti-CD28 (Clone 15E8 [IgG1 isotype]	Sanquin	N/A
Carboxyfluorescin diacetate succinumydyl ester (CFSE)	ThermoFisher Scientific	Cat#34554
Chloroacetamide (C_2_H_4_ClNO)	Sigma-Aldrich	Cat#C0267
Cytochalasin B	Sigma-Aldrich	Cat#C6762
Vybrant™ DiD cell-labeling solution	Thermo Scientific	Cat#V22887
Human Anti-GD2 Antibody Dinutuximab (Clone ch14.18)	United Therapeutics	N/A
Dihydrorhodamine (DHR)	ThermoFisher Scientific	Cat#D632
Dithiothreitol (DTT)	Sigma-Aldrich	Cat#D0632
DQ-BSA green	ThermoFisher Scientific	Cat#D12050
Fetal Calf Serum (FCS)	Bodinco	Cat#BDC-16975; Lot#20120217
FMS-like tyrosine kinase 3 ligand (Flt-3L)	Stem Cell technologies	Cat#78137
Granulocyte-macrophage colony stimulating factor (GM-CSF)	PeProTech	Cat#300-03
Granulocyte colony-stimulating factor (G-CSF)	Neupogen, clinical grade	N/A
Halt protease and phosphatase inhibitor cocktail	Pierce	Cat#78440
Herceptin/Trastuzumab	Roche	N/A
Human serum albumin (200g/L)	Sanquin	Lot#24D26H163A
Hoechst 33342	Invitrogen	Cat#H3570
Interleukin 3 (IL-3)	Stem Cell Technologies	Cat#78194
Lipopolysaccharide (LPS) isolated from *E. coli* strain O55:B5	Sigma-Aldrich	Cat#L2880
Lipopolysaccharide Binding Protein (LPB)	R&D Systems	Cat#870-LP-025
N-formyl-methionyl-leucyl-phenylalanine (fMLP)	Sigma-Aldrich	Cat#F3506
Human Stem cell growth factor (hSCF)	Produced in house (Sanquin)	N/A
Tumor necrosis factor α (TNFα)	PeProTech	Cat#300-01A
Triton X-100	Sigma-Aldrich	Cat#T8787
Phorbol 12-myristate 13-acetate (PMA)	Sigma-Aldrich	Cat#P8139
Platelet Activation Factor (PAF)	Sigma-Aldrich	Cat#P4904
Pam3Cys	EMC Microcollections	Cat#L2000
Sodium deoxycholate (C_24_H_39_NaO_4_)	BioWorld	Cat#40430018
Trifluoroacetic acid (CF_3_CO_2_H)	Biosolve	Cat#16369656
Tris(2-carboxyethyl)phosphine (TCEP) (C_9_H_12_O_6_P)	Thermo Scientific	Cat#77720
TrypLE select	Gibson	Cat#12563011
Trypsin gold	Promega	Cat#V5280
Zymosan	Santa Cruz Biotechnology	Cat#sc-258367

**Critical commerical assays**		

CD16 Microbeads, human	Miltenyi Biotec	Cat#130-045-701
Pan T cell Isolation Kit, human	Miltenyi Biotec	Cat#130-096-535

**Deposited data**		

Raw and processed proteomics data	This paper	PRIDE: PXD060579
Raw Bulk RNAseq data	This paper	GEO: GSE300707
Original code for transcriptomics, proteomics and WGCNA analysis	GitHub	www.github.com/FelipeRojasR/Neutrophils_SLII

**Experimental models: Cell lines**		

Human Neuroblastoma Cell Line LAN1	Leibniz Institute	
Human Breast Cancer Cell Line SKBR3	ATCC	

**Experimental models: Organisms/strains**		

*Candida albicans*		Strain SC5314

**Software and algorithms**		

FlowJo 10	FlowJo LCC	https://www.flowjo.com
Graphpad	Prism	https://www.graphpad.com
Rstudio	Rstudio Team, 2016	https://rstudio.com
py_diAID		https://github.com/MannLabs/pydiaid
DiaNN 1.8.1		https://github.com/vdemichev/DiaNN
Cytoscape 3.10.3		https://www.cytoscape.org/

**Other**		

CANTO II	BD	N/A
Evosep One liquid chromatography	EvoSep	N/A
TimsTOF HT mass spectrometer	Bruker	N/A
Granule Protein Annotations	Rørvig et al.^37^	http://doi.wiley.com/10.1189/jlb.1212619
MitoCarta 2.0 database		N/A
Thermomixer comfort	Eppendorf	N/A
InfinitePro2000 platereader	Tecan	N/A
ImageStream Mark II	Merck	N/A

### Experimental model and study participant details

#### Human blood donors

Blood samples as primary material were collected from anonymized healthy volunteers in accordance with Dutch regulations following the Declaration of Helsinki. Participants provided written informed consent approved by Sanquin internal Ethical Advisory Board (Privacy Declaration 1909-0176). All donors were adults older than 18 years of age, with ages ranging between 22 and 65 years of age at the time of blood donation and gender was evenly distributed among all donors. Donations were performed between 2023 and 2024, and all blood samples were processed on the same day of collection to preserve sample integrity. In total we included 10 individual donors for HSPCs and 20 for PMNs. To minimize redundancy, each experiment preferably included samples from distinct donors. The number of donors utilized per experiment is indicated in the corresponding figure legend.

#### Human model of neuroblastoma and breast cancer

The human neuroblastoma cell line LAN-1 (source: female) and the Her2Neu-positive human breast cancer cell line SKBR3 (source: female) were routinely cultured in IMDM medium supplemented with 20% FCS, 2 mM L-glutamine and 100 μg/mL streptomycin at 37°C and 5% CO_2_.

### Method details

#### Blood collection and cell isolation

Heparinized blood samples from healthy donors were diluted 1:1 with PBS containing 10% (v/v) trisodium citrate (TNC) and layered on isotonic Percoll (Pharmacia, Uppsala, Sweden, 1.076 g/mL) to be centrifuged for 20 minutes, 800 x g at room temperature. The ring, or interphase, fraction was harvested to obtain peripheral blood mononuclear cells (PBMCs). PBMCs were used to isolate T cells by magnetic-activated cells sorting (MACS) using the Pan T cell isolation kit from Miltenyi-Biotec (Bergisch Gladbach, Germany) according to the manufacturers protocol. To obtain neutrophils, ice-cold erythrocyte lysis buffer (150 mM NH_4_Cl, 10 mM KHCO_3_, and 0.1 mM EDTA) was used to lyse erythrocytes from the pellet at 4°C. Finally, neutrophils were resuspended in HEPES buffered saline solution (132 mM NaCl, 20 mM HEPES, 6.0 mM KCl, 1.0 mM MgSO_4_, 1.0 mM CaCl_2_, 1.2 mM potassium phosphate) with the addition of 5.5 mM glucose and 0.5% (w/v) human serum albumin pH 7.4 (HEPES+ medium) at 5·10^6^ cells/mL.

#### Culturing and differentiation of CD34^+^ HSPCs towards neutrophils

Hematopoietic stem cells were isolated from G-CSF mobilized peripheral blood (MPB) of healthy donors or patients after giving informed consent. HSPCs were isolated based on CD34^+^ MACS according to manufacturer's protocol, with an additional step where the isolated fraction of the first column is applied over a second MACS column. The purity of CD34^+^ cells reached above 99.6% for all vials as measured by flow cytometry. Cells were resuspended in freezing medium (IMDM, 20% fetal calf serum, 5% DMSO) and stored in liquid nitrogen until further use.

For culturing, CD34^+^ HSPCs were thawed, and DMSO was diluted by adding 20 mL ice-cold IMDM with 20% FCS dropwise to the cell suspension, which was subsequently centrifuged at 450 x g for 5 minutes at 4°C. Cell pellets were washed with ice-cold IMDM with 20% FCS and centrifuged at 450 x g for 5 minutes at 4°C. Afterwards, pellets were resuspended and brought to a concentration of 0.5·10^6^ cells/mL, either in complete IMDM (Gibco, Life Technologies, Carlsbad, CA, USA) supplemented with 10% (v/v) fetal calf serum (Bodinco, Alkmaar, The Netherlands), 10^4^ U/mL penicillin (Sigma-Aldrich), 10 ng/mL streptomycin (Sigma-Aldrich), 200 mM glutamine (Sigma-Aldrich, St. Louis, MO) or SL-II medium (Sigma) supplemented with 10^4^ U/mL penicillin and 10 ng/mL streptomycin. For the first 3 days of the culture in both media hSCF (500 ng/mL, Sanquin, in-house production), Flt-3L (250 ng/mL, Stem Cell Technologies, Vancouver, CA), IL-3 (10 ng/mL, StemCell technologies, Vancouver, CA), GM-CSF (10 ng/mL, Peprotech EC, London, UK) and G-CSF (30 ng/mL, Neupogen, clinical grade) were added, and cells were incubated at 37°C, 5% CO_2_. On day 3, 7, 10, 14 medium was refreshed and only G-CSF was used as a supplement from then on. From day 10, cells were brought to a concentration of 1·10^6^ cells/mL. On day 10, 14 and 17 cells were taken out of the culture for analysis. On day 14 and 17, cells were sorted based on a CD16^+^ MACS (Miltenyi-Biotec, Bergisch Gladbach, Germany) to obtain the most mature neutrophil population.

#### Sample preparation for mass spectrometry analysis

Purified neutrophils from blood and CD34^+^ HSPC-derived neutrophils (CD16^+^ and Bulk) were washed twice with PBS at 300 x g. The pellet was kept in a Low-binding Eppendorf tube, frozen in liquid nitrogen and stored at –80°C. For all fractions, 6 replicates were obtained. Further preparation of the mass spectrometry samples was done as described previously.^72^ In brief, cell pellets were lysed in 1% sodium deoxycholate (Bioworld), 10 mM Tris(2-carboxyethyl)phosphine (TCEP) (Thermo Scientific), 40 mM chloroacetamide (Sigma-Aldrich), 100 mM Tris-HCl pH 8.0 (Gibco) supplemented with Halt protease and phosphatase inhibitor cocktail (Thermo Scientific). Lysates were incubated for 5 minutes at 95°C and sonicated (Branson model 2510) for 10 minutes, after which trypsin gold (Promega) was added in a 1:50 (w/w) protein ratio and digested overnight at 25°C.

#### Mass spectrometry data acquisition and processing

Tryptic digests were acidified with 10% trifluoroacetic acid (Biosolve) and centrifuged at 20,000 x g for 10 minutes, supernatants were transferred to an Evotip Pure (Evosep) according to manufacturer’s guidelines and separated on a 8 cm × 150 μm, 1.5 μm Performance Column (EV1115, EvoSep) with an Evosep One liquid chromatography system (Evosep) with the 60 samples per day program. Peptides were ionized and introduced electrosprayed into a timsTOF HT mass spectrometer (Bruker). Data was acquired in dia-PASEF mode, with a 100-1700 m/z MS1 scan range. For MS2 acquisition 32 py_diAID optimized windows were used, with a 1.8 second cycle time, a 400.2-1500.8 m/z mass charge range, and ion mobility range of 0.70-1.50 1/k0. The collision energy was 20.00 eV at 0.6 1/k0 and 59 eV at 1.60 1/k0.

#### Sample preparation for RNA sequencing

Purified neutrophils from blood and CD34^+^ HSPC-derived neutrophils (CD16^+^ and Bulk) were washed thrice with ice-cold PBS and resuspended in RLT+ buffer (Qiagen, Cat nr. 52304). Samples were then immediately stored at -80°C. Cell pellets were lysed in 600 μl of RNA Plus buffer (Qiagen RNeasy plus kit) supplemented with 1% of mercapto-ethanol. The volume of resuspended cells was homogenized with QiaShredder Columns and isolated with the Qiagen RNAeasy plus kit according to manufacturer’s protocol. The RNA was eluted with 40 μL of nuclease free water. RNA concentrations between 10 and 100 ng/μL were obtained. For RNA-Seq using rRNA reduction (total transcriptome) optimal total input of ≥ RNA 250 ng per sample was required to allow for QC and normalization with sample volumes greater than 20 μL. RNA quality for all samples were RIN/RQN ≥ 7.

#### RNA sequencing data acquisition and library preparation

The RNA was sent to GenomeScan to perform RNA sequencing (rRNA depleted) of 46 total RNA samples (human), using Illumina NovaSeq sequencing, Paired-End, 150 bp. Briefly, the NEBNext Ultra II Directional RNA Library Prep Kit for Illumina was used to process the samples. Samples were prepared according to the protocol "NEBNext Ultra II Directional RNA Library Prep Kit for Illumina" (NEB #E7760S/L). rRNA was depleted from total RNA using the Qiagen fast select kit (#334387). After fragmentation of the rRNA reduced RNA, a cDNA synthesis was performed. This was used for ligation with the sequencing adapters and PCR amplification of the resulting product. The quality and yield after sample preparation was measured with the Fragment Analyzer. Clustering and DNA sequencing using the NovaSeq6000 was performed according to manufacturer's protocols. A concentration of 0.8 nM of DNA was used. Per sample ∼9 Gb, 30 million Paired-End reads were obtained.

#### Flow cytometric surface marker analysis

All antibodies used for cell surface expression analysis of neutrophils are listed in the key resources table. Markers to discriminate the different developmental stages of CD34^+^ HSPC-derived neutrophils (CD15, CD11b and CD16) were taken along in the FACS analysis. 50,000 cells were used for cell surface marker labeling. Cells were incubated for 15 minutes with antibodies at 4°C, and subsequently washed with PBS + 0.5% human serum albumin at 4°C, 300 x g. The Canto II flow cytometer together with FlowJo software (version 10.8.1) were used for analysis.

#### May-Giemsa stained cytospins

50,000 cultured neutrophils or PMNs were spun down on glass microscope slides during 10 min centrifugation at 1,000 RPM (Shando 4, cytospin, Thermo). Slides were air-dried, stained for 5 minutes in May-Grünwald (Merck), washed with deionized water followed by 30 minutes Giemsa (Merck) staining. Slides were rinsed again with demi-water, air-dried and analyzed by the Zeiss Scope.A1 microscope using a 50x oil objective.

#### Functional assays

##### Adhesion

Cells were labelled with Calcein AM at 4 μg/mL for 30 minutes at 37°C and subsequently washed twice with PBS, 300 x g and resuspended in HEPES+ medium. Per condition, 160,000 calcein-labelled neutrophils were added to an uncoated 96-wells maxisorp plate (Thermofisher). Different stimuli were added to the wells in the plate (HEPES+, G-CSF (2 ng/mL), DTT(10 mM), LBP/LPS (250 ng/mL; 50 ng/mL), fMLP (1μM, Sigma-Aldrich, St. Louis, MO, USA), C5a (100 nM), PMA (0.1 ng/mL) and incubated for 30 minutes at 37°C and 5% CO_2._ Subsequently, the plate was stroke out and washed twice with PBS before leaving it to dry. To lyse the cells, 100 μl 0.5% Triton X-100 (TX-100) (Sigma-Aldrich) was added for 2-5 minutes before measuring by the infinitiPRO2000 plate reader. To determine what percentage of cells attached to the plate, 160,000 calcein-labeled cells were added to the plate with 0.5% TX-100 and directly measured by the plate reader as control. All values were calculated with the following formula: % adherent cells = (RFU sample/RFU control)·100%.

##### Chemotaxis

Cells were labelled with Calcein AM at 4 μg/mL for 30 minutes at 37°C and subsequently washed twice with PBS, 300 x g and resuspended in HEPES+ media to 2·10^6^ cells/mL. Either C5a (10 nM, Peprotech) or fMLP (100 nM, Peprotech) were used as a chemoattractant for measuring neutrophil chemotaxis. As a negative control, HEPES+ was added to a 24-wells plate and pre-incubated for 5 minutes at 37°C. Subsequently, 600,000 calcein-labelled cells were added to a FluoroBlock insert. Inserts were placed into the 24-wells plate and chemotaxis was measured by the infinitePRO2000 platereader (Tecan, Mannedorf, Switzerland). Data are shown as the maximum slope of relative fluorescent units (RFU)/min measured.

##### Taxiscan

The insert was prepared according to manufacturer's protocol using a 5-micron chip and HEPES+ media. After preheating the plate, the insert was heated to 37°C, surplus media removed, and either PMNs or cultured neutrophils were inserted and lined on the chip. 2 mL of HEPES+ media was added, followed by fMLP or HEPES+ as negative control. Images were taken every 30 seconds for 1 hour. Images were then converted to videos using FIJI (version 1.53).

##### Reactive oxygen species (ROS) production

ROS production by the NADPH oxidase complex was measured using the Amplex Red kit (Molecular Probes, Life Technologies, Carlsbad, CA, USA) according to the manufacturer's instructions. Neutrophils (1·10^6^ cells/mL) were pre-incubated in HEPES+ medium for 5 minutes at 37°C, in the presence of Amplex Red (0.5 μM) and horseradish peroxidase (1 U/mL), and subsequently stimulated with serum opsonized *E. coli* (OD_600_ = 0.25), Zymosan (1 mg/ml), Serum-treated Zymosan (STZ) (1 mg/ml), PMA (100 ng/ml) and Platelet Activating Factor (PAF)/fMLP (both at 1 μM final concentration). Fluorescence was measured every 30 seconds for 30 minutes in the HTS7000+ plate reader (Tecan, Zurich, Switzerland). A calibration curve of hydrogen peroxide ranging from 0 to 1 μM was used to determine the concentration of peroxide during the experiment. The maximal slope of hydrogen peroxide release was calculated over 2-minute intervals.

##### Phagocytosis

Cells were labelled with Calcein red-orange at 0.8 ng/mL for 30 minutes at 37°C and subsequently washed twice with PBS, 300 x g and resuspended in HEPES+ media. Zymosan particles were thawed and subsequently sonicated in a water bath for 10 minutes. Supernatant was taken and labelled with FITC isothiocyanate at 1 μg/mL for 30 minutes at 37°C shaking whereafter washed thrice with PBS, 10,000 x g and resuspended in PBS. For opsonization, FITC-labelled zymosan particles were incubated with 10% serum for 10 minutes at 37°C shaking. Subsequently, calcein red-orange labelled neutrophils were incubated with either bare or serum-opsonized FITC-labelled zymosan particles at 450 RPM at 37°C (Thermomixer Comfort) in Eppendorf tubes. At different time points, samples were taken and fixed (20 mM NaF, 0.5% PFA, 1% BSA in PBS). Samples were then measured either by the Canto II flow cytometer or ImageStream Mark II (Merck) for uptake of zymosan particles. Data were analyzed by FlowJo software (version 10.8.1) and using the Software program IDEAS®.

##### Killing

Cultures of *E. coli* and *S. aureus* were made by growing 1 ml of frozen stock (PBS + 10% glycerol) with OD_600_ = 0.5 in 19 ml of LB for 2-4 hours at 37°C, 200 RPM (Innova 44, New Brunswick Scientific). Pre-cultures of *C. albicans* were grown overnight by adding 1 ml frozen stock (PBS + 10% glycerol) with OD_625_ = 0.5 to 19 ml of LB+kanamycin at 37°C, 200 RPM. Cultures were grown with 1 ml pre-culture in 19 ml LB+kanamycin for 2-4 hours on the day of the assay. Pathogens were washed thrice with PBS at 3750 x g (Rotanta 380), 5 minutes at RT.

Pathogens were put in HEPES+ at OD_600_ = 1.00. Bacteria and *C. albicans* were then opsonized with 10% plasma at OD=0.1 and OD=0.2 respectively for 10-15 minutes at 37°C, 650 RPM in a thermoshaker (Thermomixer Comfort, 1.5 mL eppendorf insert, Eppendorf). For PMNs and cultured neutrophils, 400 μl of cell suspension at 5·10^6^ cells/mL was pre-incubated for 5 minutes at 37°C, 650 RPM in a thermoshaker with 90 μl of HEPES+ or opsonized bacteria. For *C. albicans*, 100 μl of HEPES+ was pre-incubated for 5 minutes at 37°C, 650 RPM in a heat block for timepoint t=0, and 100 μL of PMN or cultured neutrophil suspension at 5·10^6^ cells/mL were pre-incubated at 37°C, 650 RPM in a heat block. After pre-incubation and opsonization, 10 μl of opsonized bacteria was added to the 490 μl cell suspensions, and 25 μl of unopsonized or opsonized *C. albicans* were added to the 100 μl HEPES+ or cell suspensions. For bacteria, at each timepoint (*E. coli* t=0, 5, 10, 15 and 30 minutes; *S. aureus* t=0, 5, 15, 30 and 60 minutes) 50 μl of cell suspension was added to 2.5 mL of H_2_O with pH=11.0 and subsequently vortexed. For *C. albicans*, 100 μl of sample was taken and added to 2.5 mL of filtered H_2_O with pH=11.0 and subsequently vortexed at t=0 and t=120 minutes. After 5 minutes, samples were vortexed, and 50 μl of alkaline cell suspension was transferred to 450 μl of PBS for bacteria and vortexed, and for *C. albicans*, 100 μl of alkaline cell suspension was transferred to 400 ul of PBS and vortexed. For each time point, 100 μl of PBS cell suspension was plated on 10-cm LB+agar plates. Colonies were counted the following day. Killing was determined as the inverse of the %CFU remaining compared to t=0 for each pathogen.

##### Chromium-based antibody dependent cellular cytotoxicity (ADCC)

The radioactive chromium-based ADCC assay was performed as described before (Martínez-Sanz et al., 2021). Briefly, 1·10^6^ target cells (LAN-1 and SKBR3) were labeled with 100 μCi ^51^Cr (PerkinElmer, Waltham, MA, USA) for 90 minutes at 37°C. Afterwards, the cells were washed thrice with PBS before resuspending them in complete IMDM. The chromium-labeled target cells were incubated in a 96-well U-bottom plate (Corning) with effector cells (PMNs or CD34^+^ HSPC-derived neutrophils) using a target:effector ratio of 1:50 (i.e. 5,000 cells:250,000 cells) in the presence or absence of the corresponding antibody (1 μg/mL Dinutuximab (Unituxin, Ch14.18, United Therapeutics) for LAN-1 and 1 μg/mL Trastuzumab (Roche) for SKBR3). For the GM-CSF stimulation of PMNs, GM-CSF (10 ng/mL, Peprotech) was added to the cells and incubated for 30 minutes, shaking at 37°C in a waterbath before starting the co-culture. After co-culturing target and effector cells for a period of 4 hours at 37°C and 5% CO_2_, cells were spun down (300 x g for 5 minutes) and 30 μl supernatant was transferred to a Lumaplate (PerkinElmer). The plate was dried overnight at RT before analyzing in a MicroBeta^2^ plater reader (PerkinElmer). The percentage of cytotoxicity was calculated using the following formula: (experimental counts per minute (CPM)-spontaneous CPM)/(maximum CPM-spontaneous CPM)·100%. Spontaneous cell death was determined by incubating chromium-labeled target cells with medium only and maximum cell death was determined by treating chromium-labeled target cells with a 0.1% Triton X-100 solution. All conditions were performed in duplicate.

##### T cell proliferation

To compare immunosuppressive capacity of cultured neutrophils to PMNs, a T cell proliferation assay was performed. Purified T cells were labeled with CFSE cell proliferation dye (Molecular probes, Life Technologies, Carlsbad, CA, USA) for 8 minutes, shaking in a waterbath at 37°C and washed twice. After labeling, T cells were cocultured with either PMNs or SL-II CD16^+^ neutrophils in a 1:3 ratio (20,000 T cells and 60,000 neutrophils). Cells were resuspended in complete IMDM and plated in 96-well flat bottom plates at 37°C. T cell proliferation was induced using a combination of anti-CD3 and anti-CD28 monoclonal antibodies in suspension (5 μg/ml), whereas neutrophils were activated using TNFα (10 ng/mL, Peprotech EC, London, UK). Both CD3/28 and TNFa were added as soluble factors in the medium of the co-culture. After 4 days, T cell proliferation was measured by flow cytometry based on CFSE dilution. The formation of damaged T cells was determined based on forward/side scatter. To assess differential effects on CD4^+^ and CD8^+^ T cells, CD4-APC and CD8-PerCP-Cy5.5 were used for flowcytometric staining. To stain PMNs and cultured neutrophils, CD15-PE was used.

##### Trogocytosis of T cells by neutrophils

T cells were labeled with lipid-intercalating dye DiD (Thermo Scientific) for 30 minutes in a 37°C waterbath and washed twice with PBS before they were added to a 96-well flat bottom plate together with calcein red-orange labeled neutrophils in a 1:3 ratio (100,000 T cells and 300,000 neutrophils). Anti-CD3/CD28 monoclonal antibodies (5μg/ml) and TNFα (10ng/mL) were added in suspension and used to stimulate T cells and neutrophils respectively. After 180 minutes, cells were resuspended and fixed, whereafter analyzed by flow cytometry and ImageStream using FlowJo software (version 10.8.1) and software program IDEAS® accordingly.

For Live Cell Imaging of SL-II CD16^+^ neutrophils, DiD labeled T cells were incubated with calcein red-orange labeled neutrophils with the addition of anti-CD3/CD28 monoclonal antibodies (5μg/ml) and TNFα (10ng/mL) for 4h in 8-well Ibidi chambers (Ibidi, Gräfelfing, Germany) in a T:E ratio of 1: 3 (15.000 T cells and 45.000 neutrophils). Co-cultures were imaged in the LSM 980 Airyscan 2 (Zeiss, Oberkochen, Badem-Württemberg, Germany). Data processing was performed using Imaris Viewer 10.1 (Oxford Instruments, Oxfort, UK).

##### Protease release

Release of active Cathepsin G (CTSG) and Elastase (ELANE) was measured by changes in fluorescence due to digestion of the probe DQ-BSA. 250,000 neutrophils together with DQ-BSA reagent (10μg/mL) and either HEPES+ or CytoB (5 μg/mL) were added per well in a white 96-wells plate and pre-incubated for 5 minutes at 37°C in a plate reader. Cells primed with CytoB were subsequently stimulated with fMLP (1 μM). As a positive control cells were lysed with TX-100. Fluorescence was measured over 60 minutes every 30 seconds and protease activity was calculated as maximum slope per minute over 2 minutes.

##### Degranulation

Primary (azurophilic) granule release and secondary (specific) granule release were measured after stimulation with PAF/fMLP or CytoB/fMLP, respectively. Thereafter, neutrophils were put at 2.5·10^6^ cells/mL and were first pre-incubated with PAF (1 μM) or CytoB (5 μg/ml) for 5 minutes at 450 RPM at 37°C (Thermomixer Comfort, Eppendorf) in Eppendorf tubes. After priming, cells were stimulated with fMLP (1 μM) for 10 minutes at 450 RPM at 37°C. After stimulation, cells were put on ice and stained with granule markers CD63, CD66b and LOX-1. Markers to discriminate the different developmental stages of CD34-derived neutrophils (CD15, CD11b and CD16) were taken along in the FACS analysis. Data are expressed as gMFIs and background staining was subtracted.

### Quantification and statistical analysis

#### Mass spectrometry data analysis

The raw mass spectrometry data files were processed using the DiaNN software (version 1.8.1), proteins and peptides were detected by querying the filtered human Swissprot database (release 2021.22.04). Standard settings were used, using a generated library based spectra search. Maximum number of variable modifications was set at 1, Protein Interference used was “Protein names (from FASTA)” and quantification strategy “Robust LC (high accuracy)”. For further analysis detected proteins were filtered for proteotypic and ≥2 unique peptides per protein and proteins should be quantified in 75% of samples in at least one condition. LFQ values were log2 transformed and missing values were imputed by normal distribution (width = 0.3, downshift = 1.8), assuming these proteins were close to the detection limit. Principal component analysis was performed on un-imputed log2LFQ. Statistical significance was determined with moderated t-test using the limma package applying FDR <0.05 and |log2 fold change| > 1 as thresholds.

#### RNA-sequencing data analysis

Reads were trimmed for the first 10 base pairs displaying nucleotide distribution imbalances using cutadapt.^73^ Reads with a minimal required length of 36bp with a right window of 4bps, average window quality of at least 15, removal of adapters, filtering for low complexity reads, trimming for poly X and poly G and deduplication of pair end reads with a minimal deduplication accuracy of 4 using FASTP.^74^ Reads were mapped to the GRCh38 human genome (release 111) with STAR^75^ using two-pass mode. Mapped reads were deduplicated using UMI tools^76^ removing chimeric pairs and unpaired reads. Next, a read count matrix was obtained using featureCounts.^77^ Genes with a low number of reads were filtered using two different criteria. First, transcripts with a count per million lower than 1 in less than 70% of the samples in at least one condition were excluded. Next, transcripts with a mean count per million lower than 1 across all conditions were also excluded. Differential expression between any two groups was done using DeSeq2^78^ without any beta priors and using the ASHR method for log2 fold change shrinkage applying FDR <0.05 and |log2 fold change| > 2 as thresholds. Differential expression was performed accounting for experimental conditions pooling replicates together without any additional covariates. Gene counts were normalized using the variance stability transformation (VST) blinded to the experimental design using DeSeq2 for visualization purposes and further analyses, detailed below. Transcripts ensemble gene identifiers were annotated to HUGO symbols using the Genome Wide Annotation for Human (org.Hs.eg.db, version 3.8.2) in R.

#### Integrative data analysis of the proteome and transcriptome

To combine these datasets, the transcriptome was limited to only include transcripts for which there was a protein available matching gene symbol. For each comparison, the VST normalized counts and imputed log2(LFQ) were transformed to z-scores independently and used to construct heatmaps. For these, heatmaps enrichment was performed using an overrepresentation analysis limiting the transcript/protein entries to the maximum combined set of transcripts or proteins independently across comparisons. For pairwise comparisons, gene set enrichment analysis (GSEA) was performed on ranked log2fold for transcripts and proteins separately using ClusterProfiler^79^ with a gene set size ranging between 20 to 400 using Gene Ontology,^29^ Wiki-Pathways^27^ and Reactome^28^ databases. Gene sets and pathways were considered significant at a p value of < 0.05. Scatterplots comparing the effect sizes between transcriptome and proteome selecting for day 17 CD16^+^ neutrophils compared to PMNs were produced by aggregating all transcript/protein pairs together highlighting those considered to be significant (FDR <0.05 and |log2 fold change| > 2 for the transcriptome and |log2 fold change| > 1 for the proteome). Boxplots for specific genes were plotted using a log2 scale for VST normalized read counts as well as LFQ values.

Proteins suspected to be of foreign source in the PMN samples were identified by selecting all proteins without a matching transcript in any of the SL-II samples. Protein-protein interaction network was constructed with STRING-DB.^80^ The network was curated by removing isolated proteins or any cluster with less than four nodes using Cytoscape.^81^ This set of proteins was enriched for overrepresented molecular mechanisms related to platelets and plasma using ClusterProfiler with adjusted p value < 0.05 as threshold.

Sankey plots were used to show the relative distribution of up-regulated, down-regulated and non-statistically different transcripts and proteins across stages of maturation. For co-expression analyses, HSCs, Bulk day 10, MACS sorted CD16^+^ day 14 and MACS sorted CD16^+^ day 17 samples were included. Z-scores from the VST normalized counts and imputed log2LFQ from all transcript/protein pairs were obtained. WGCNA^34^ was constructed using a signed network with a soft power of 15. Modules were identified using a dynamic tree detection cut height of 0.9 and merge cut height of 0.1 including a minimum module size of 20 and maximum module size of 75000. Modules were combined using a person correlation threshold of 0.7. Modules were enriched using ClusterProfiler using Gene Ontology, Wiki-Pathways and Reactome databases. Manual enrichment of transcript/protein pairs of each module was performed using a fisher exact test followed by Benjamini-Hochberg multiple testing correction based on MitoCarta3.0,^35^ Recon3D^36^ and the complete dataset of neutrophil granule components.^37^ Transcript/proteins pair connectivity was used as a proxy to select the most relevant genes in each module. Connectivity was computed using the absolute number of edges corresponding to each gene in the adjacency matrix, excluding the diagonal. For each module we estimated the linear association between the z-scores of transcripts and proteins and the stage of maturation for which linear models were fitted, with z-scores set as outcome and time of maturation as a continuous covariate highlighting those considered to be significant with a p value < 0.05.

Statistical analysis was done using R version 4.5.0 and GraphPad Prism version 9.1.1 (GraphPad software, Boston, Massachusetts, USA). P values lower than 0.05 were considered statistically significant and were labelled as ∗p<0.05, ∗∗p<0.01, ∗∗∗p<0.001 and ∗∗∗∗ p<0.0001. Statistical details of each experiment can be found in figure legends, including the test used, number of individual donor samples (n value), p value and definitions.

## Supplemental information

Document S1. Figures S1–S6 and Tables S1, S7, and S8

Table S2. Log2fold change and P values of transcripts and proteins when comparing SL-II CD16^+^ cultured neutrophils and PMNs A. Complete list of transcripts and proteins. B. Subset of transcripts and proteins that have a higher RNA abundance and a lower protein abundance in SL-II CD16^+^ cultured neutrophils compared to PMNs. C. Effect size for transcriptome and proteome for genes known to be involved in MDSC activity when comparing SL-II CD16^+^ cultured neutrophils and PMNs.

Table S3. Gene set enrichment analysis of up- and down-regulated transcripts and proteins that were obtained when comparing SL-II CD16^+^ cultured neutrophils to PMNs Biological processes were obtained either from Wiki-Pathways (∗), Gene Onthology (∗∗) or Reactome (∗∗∗). All biological processes with an FDR <0.05 were considered statistically significant. A. Gene set enrichment values for all identified pathways. Labels represent: ∗ for wikipathways, ∗∗ for Gene Ontology and ∗∗∗ Reactome. B. List of all gene names that are included in all the enrichment analyses. C. Effect size of proteins and transcripts associated with Reactome neutrophil degranulation enrichment when comparing SL-II CD16^+^ cultured neutrophils to PMNs.

Table S4. Protein-Protein interaction network of all proteins for which no transcript was observed in either PMNs or SL-II cultured neutrophils

Table S5. Complete list of transcript/protein pairs contained in each one of the 14 modules identified using a WGCNA A. List of genes included in each one of the modules and the respective groups identified using pearson correlation B. List of module membership, gene trait and individual trait correlation values for HSPCs, SL-II bulk day 10, SL-II CD16^+^ day 14 and SL-II CD16^+^ day 17 for all transcript/protein pairs.

Table S6. Presence or absence of each of the transcript/protein pairs in Mitocarta3.0, Recon3D or the list of granule related proteins A. List of transcript/protein pairs which overlap with the genes available in mitocarta3.0, recon3D, or the complete list of granule-associated proteins. B. Values of the Fisher's exact test for each module when associated with mitocarta3.0, recon3D, or the complete list of granule-associated proteins.
