# Supplementary material for: Characterization of human CD34+ HSPC-derived neutrophils with limited myeloid-derived immunosuppressive cell activity
Source: iScience. 2025 Aug 20;28(9):113404. doi: 10.1016/j.isci.2025.113404 (PMC12496184; doi:10.1016/j.isci.2025.113404)
Supplement: Document S1. Figures S1–S6 and Tables S1, S7, and S8 [file mmc1.pdf]

iScience, Volume *28*

Supplemental information

Characterization of human CD34$^+$ HSPC-derived neutrophils with limited myeloid-derived immunosuppressive cell activity

Steven D.S. Webbers, Angela A.F. Gankema, Fleur van Oosterom, Felipe Rojas-Rodriguez, Dané S. Koops, Anna K. Klaus, Judy Geissler, Teunis J.P. van Dam, Hanke L. Matlung, Robin van Bruggen, Helin Tercan, Arie J. Hoogendijk, and Taco W. Kuijpers

Figure S1

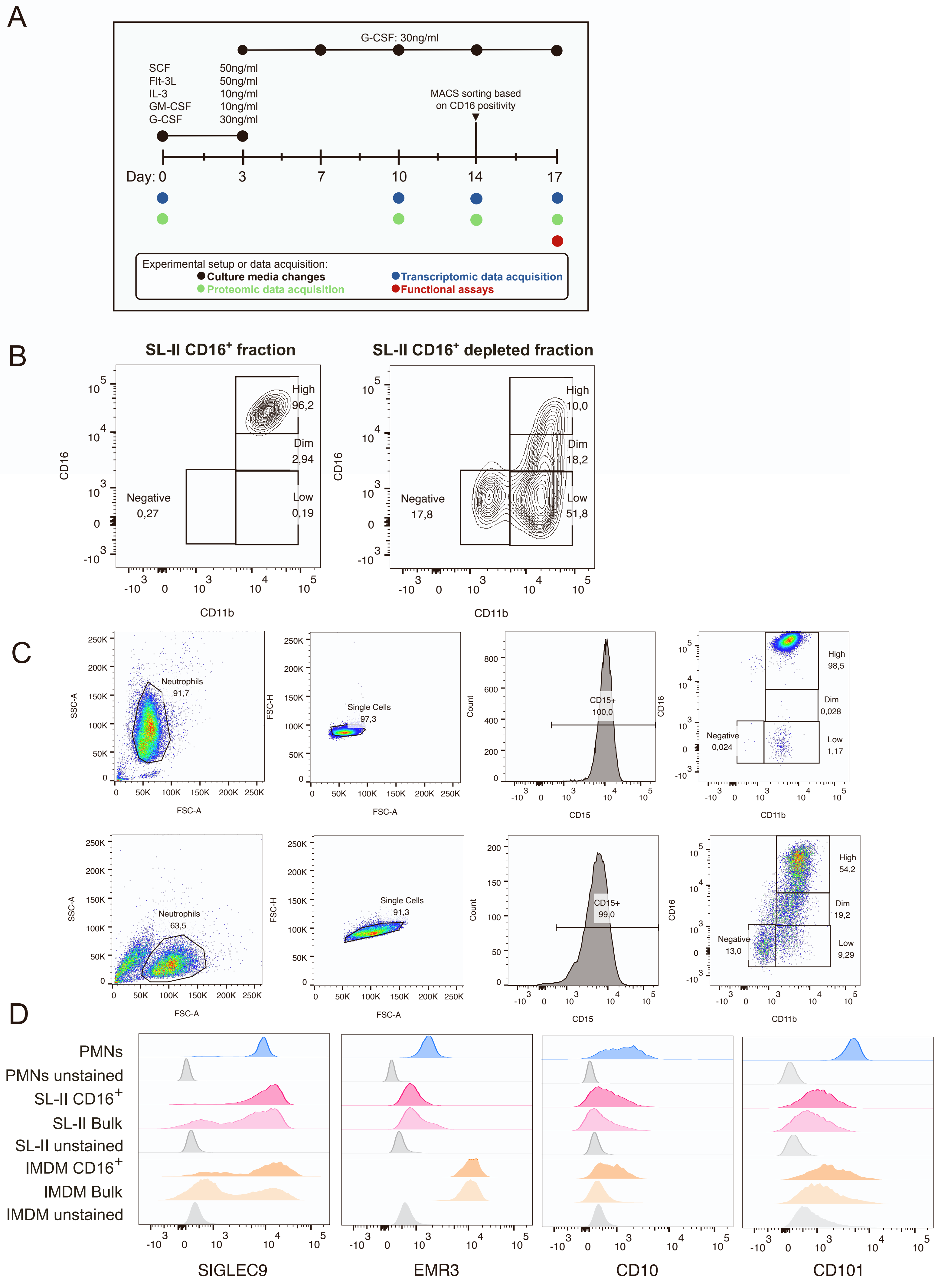
A
G-CSF: 30ng/ml
SCF 50ng/ml
Flt-3L 50ng/ml
IL-3 10ng/ml
GM-CSF 10ng/ml
G-CSF 30ng/ml
MACS sorting based on CD16 positivity
Day: 0 3 7 10 14 17
Experimental setup or data acquisition:
Culture media changes
Proteomic data acquisition
Transcriptomic data acquisition
Functional assays
B
SL-II CD16+ fraction
SL-II CD16+ depleted fraction
High 96,2
Dim 2,94
Negative 0,27
Low 0,19
High 10,0
Dim 18,2
Negative 17,8
Low 51,8
CD16
CD11b
C
Neutrophils 91,7
Single Cells 97,3
CD15+ 100,0
High 98,5
Dim 0,028
Negative 0,024
Low 1,17
Neutrophils 63,5
Single Cells 91,3
CD15+ 99,0
High 54,2
Dim 19,2
Negative 13,0
Low 9,29
SSC-A
FSC-A
FSC-H
Count
CD15
CD16
CD11b
D
PMNs
PMNs unstained
SL-II CD16+
SL-II Bulk
SL-II unstained
IMDM CD16+
IMDM Bulk
IMDM unstained
SIGLEC9
EMR3
CD10
CD101

Figure S2

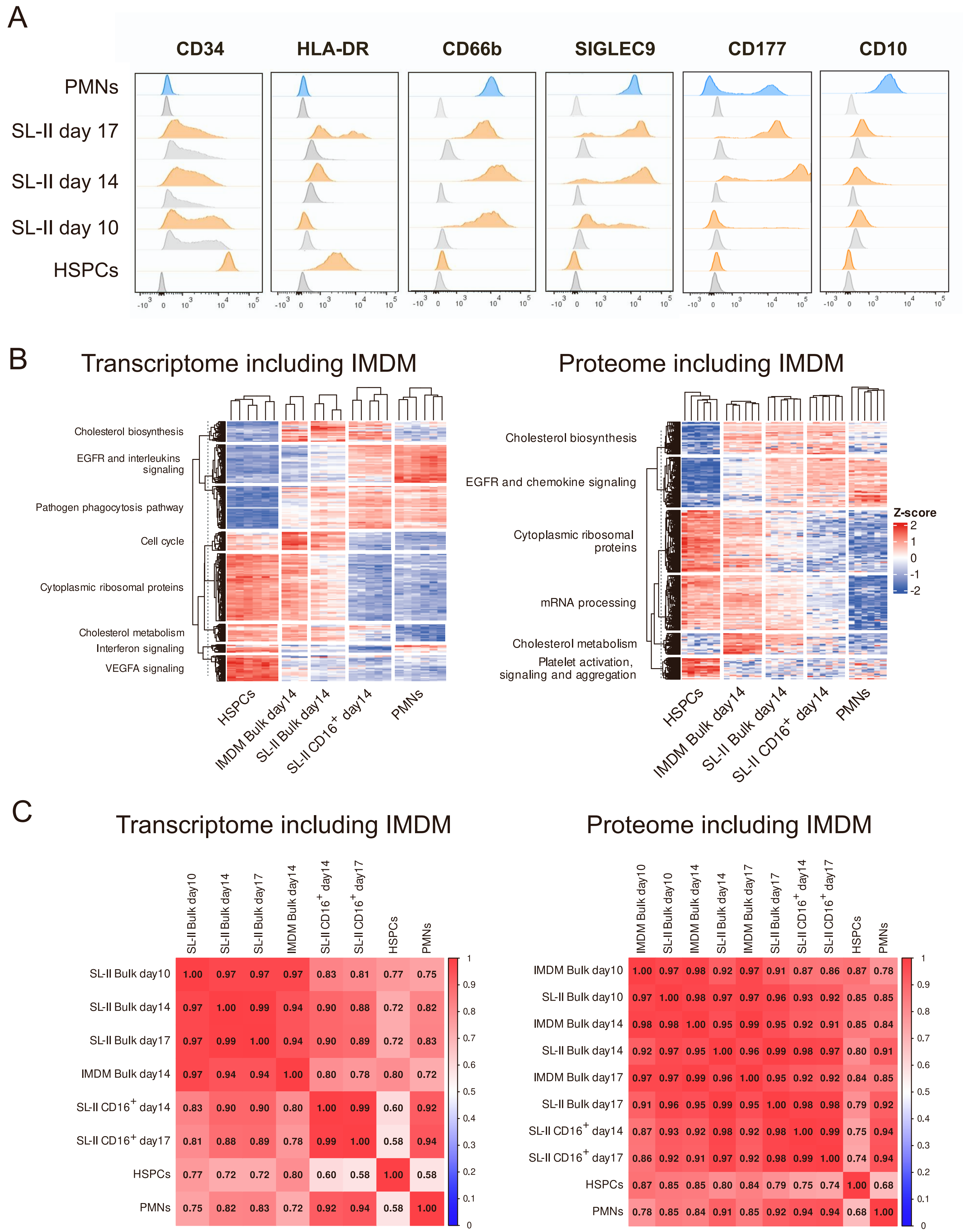
A
CD34
HLA-DR
CD66b
SIGLEC9
CD177
CD10
PMNs
SL-II day 17
SL-II day 14
SL-II day 10
HSPCs
B
Transcriptome including IMDM
Cholesterol biosynthesis
EGFR and interleukins signaling
Pathogen phagocytosis pathway
Cell cycle
Cytoplasmic ribosomal proteins
Cholesterol metabolism
Interferon signaling
VEGFA signaling
HSPCs
IMDM Bulk day14
SL-II Bulk day14
SL-II CD16+ day14
PMNs
Proteome including IMDM
Cholesterol biosynthesis
EGFR and chemokine signaling
Cytoplasmic ribosomal proteins
mRNA processing
Cholesterol metabolism
Platelet activation, signaling and aggregation
Z-score
2
1
0
-1
-2
HSPCs
IMDM Bulk day14
SL-II Bulk day14
SL-II CD16+ day14
PMNs
C
Transcriptome including IMDM
SL-II Bulk day10
SL-II Bulk day14
SL-II Bulk day17
IMDM Bulk day14
SL-II CD16+ day14
SL-II CD16+ day17
HSPCs
PMNs
SL-II Bulk day10 1.00 0.97 0.97 0.97 0.83 0.81 0.77 0.75
SL-II Bulk day14 0.97 1.00 0.99 0.94 0.90 0.88 0.72 0.82
SL-II Bulk day17 0.97 0.99 1.00 0.94 0.90 0.89 0.72 0.83
IMDM Bulk day14 0.97 0.94 0.94 1.00 0.80 0.78 0.80 0.72
SL-II CD16+ day14 0.83 0.90 0.90 0.80 1.00 0.99 0.60 0.92
SL-II CD16+ day17 0.81 0.88 0.89 0.78 0.99 1.00 0.58 0.94
HSPCs 0.77 0.72 0.72 0.80 0.60 0.58 1.00 0.58
PMNs 0.75 0.82 0.83 0.72 0.92 0.94 0.58 1.00
Proteome including IMDM
IMDM Bulk day10
SL-II Bulk day10
IMDM Bulk day14
SL-II Bulk day14
IMDM Bulk day17
SL-II Bulk day17
SL-II CD16+ day14
SL-II CD16+ day17
HSPCs
PMNs
IMDM Bulk day10 1.00 0.97 0.98 0.92 0.97 0.91 0.87 0.86 0.87 0.78
SL-II Bulk day10 0.97 1.00 0.98 0.97 0.97 0.96 0.93 0.92 0.85 0.85
IMDM Bulk day14 0.98 0.98 1.00 0.95 0.99 0.95 0.92 0.91 0.85 0.84
SL-II Bulk day14 0.92 0.97 0.95 1.00 0.96 0.99 0.98 0.97 0.80 0.91
IMDM Bulk day17 0.97 0.97 0.99 0.96 1.00 0.95 0.92 0.92 0.84 0.85
SL-II Bulk day17 0.91 0.96 0.95 0.99 0.95 1.00 0.98 0.98 0.79 0.92
SL-II CD16+ day14 0.87 0.93 0.92 0.98 0.92 0.98 1.00 0.99 0.75 0.94
SL-II CD16+ day17 0.86 0.92 0.91 0.97 0.92 0.98 0.99 1.00 0.74 0.94
HSPCs 0.87 0.85 0.85 0.80 0.84 0.79 0.75 0.74 1.00 0.68
PMNs 0.78 0.85 0.84 0.91 0.85 0.92 0.94 0.94 0.68 1.00

Figure S3

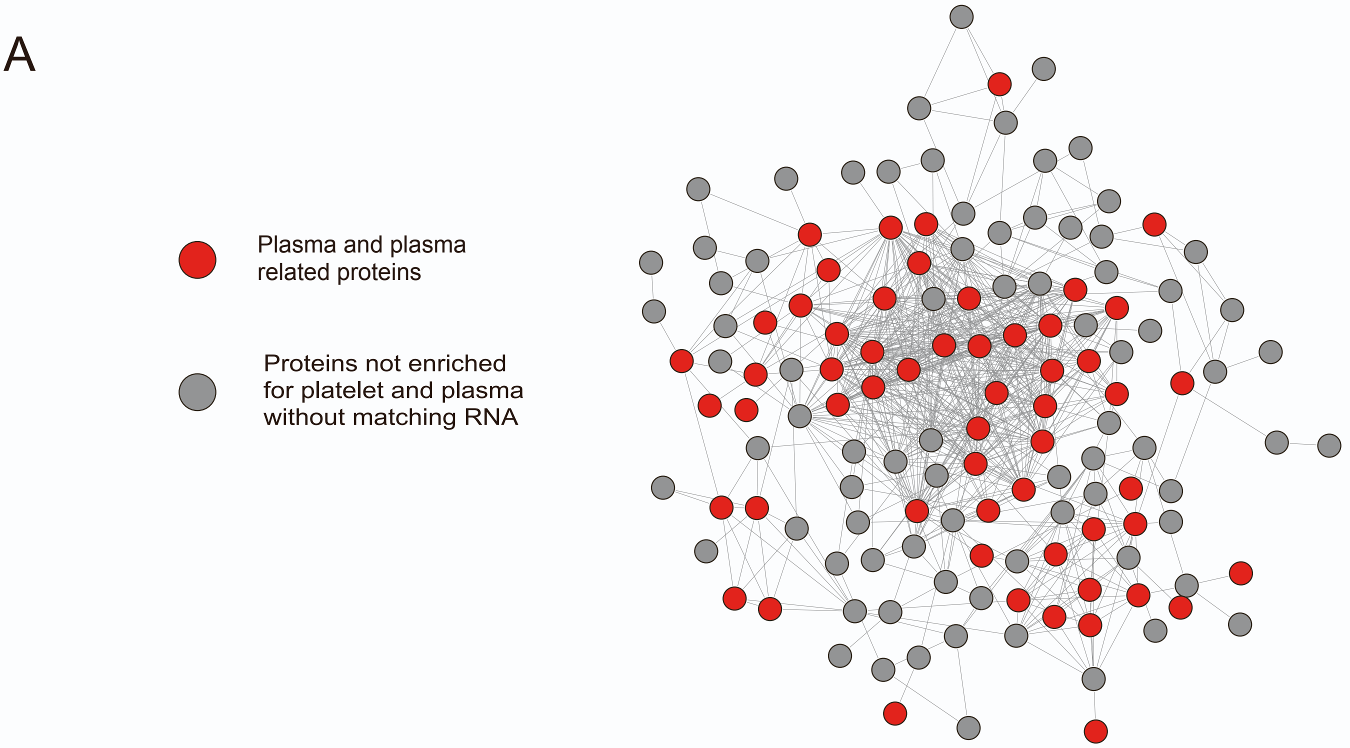
A
Plasma and plasma related proteins
Proteins not enriched for platelet and plasma without matching RNA

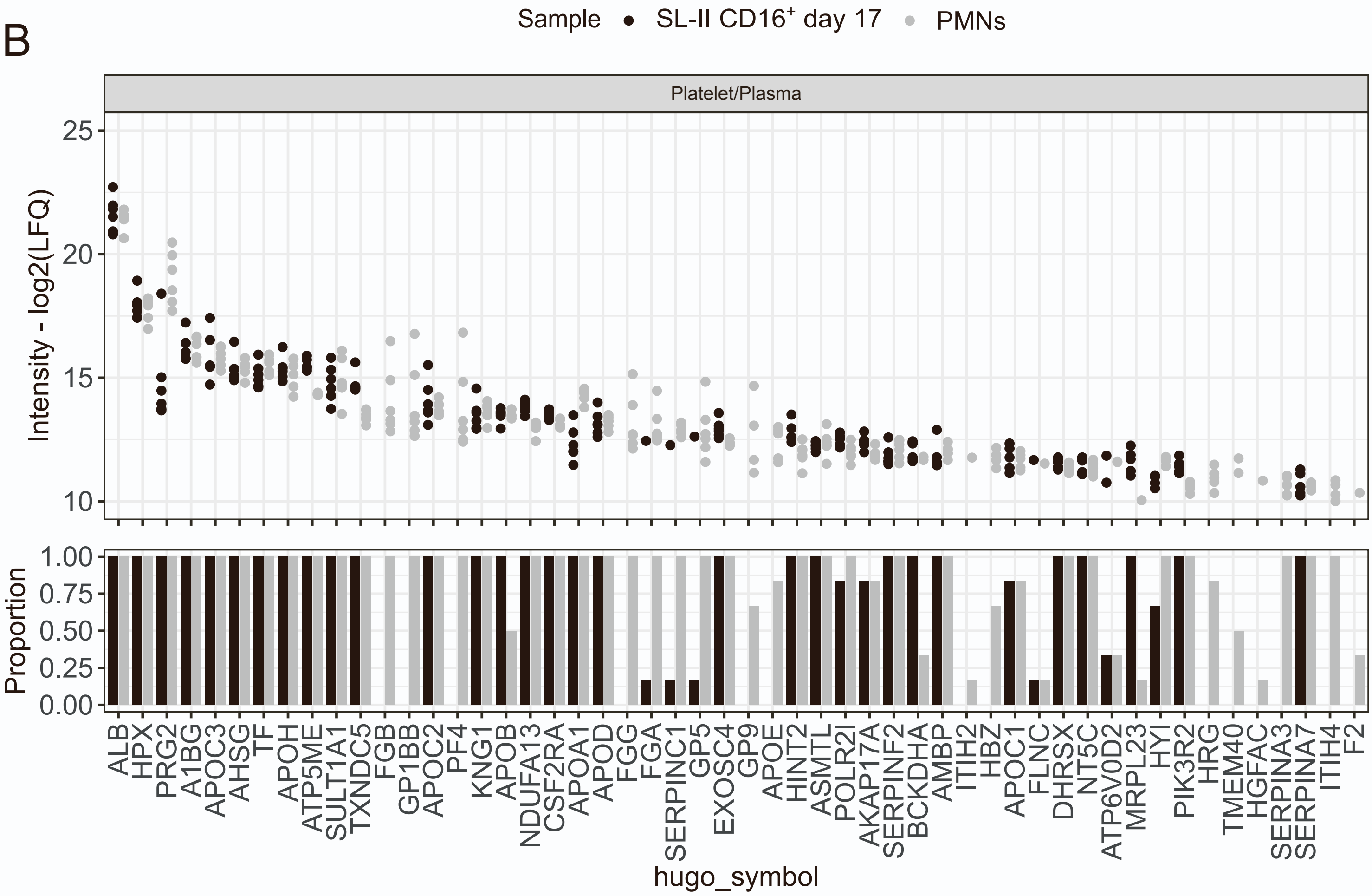
B
Sample
SL-II CD16+ day 17
PMNs
Platelet/Plasma
Intensity - log2(LFQ)
Proportion
hugo_symbol
ALB
HPX
PRG2
A1BG
APOC3
AHSG
TF
APOH
ATP5ME
SULT1A1
TXNDC5
FGB
GP1BB
APOC2
PF4
KNG1
APOB
NDUFA13
CSF2RA
APOA1
APOD
FGG
FGA
SERPINC1
GP5
EXOSC4
GP9
APOE
HINT2
ASMTL
POLR2I
AKAP17A
SERPINF2
BCKDHA
AMBP
ITIH2
HBZ
APOC1
FLNC
DHRSX
NT5C
ATP6V0D2
MRPL23
HYI
PIK3R2
HRG
TMEM40
HGFAC
SERPINA3
SERPINA7
ITIH4
F2

Figure S4

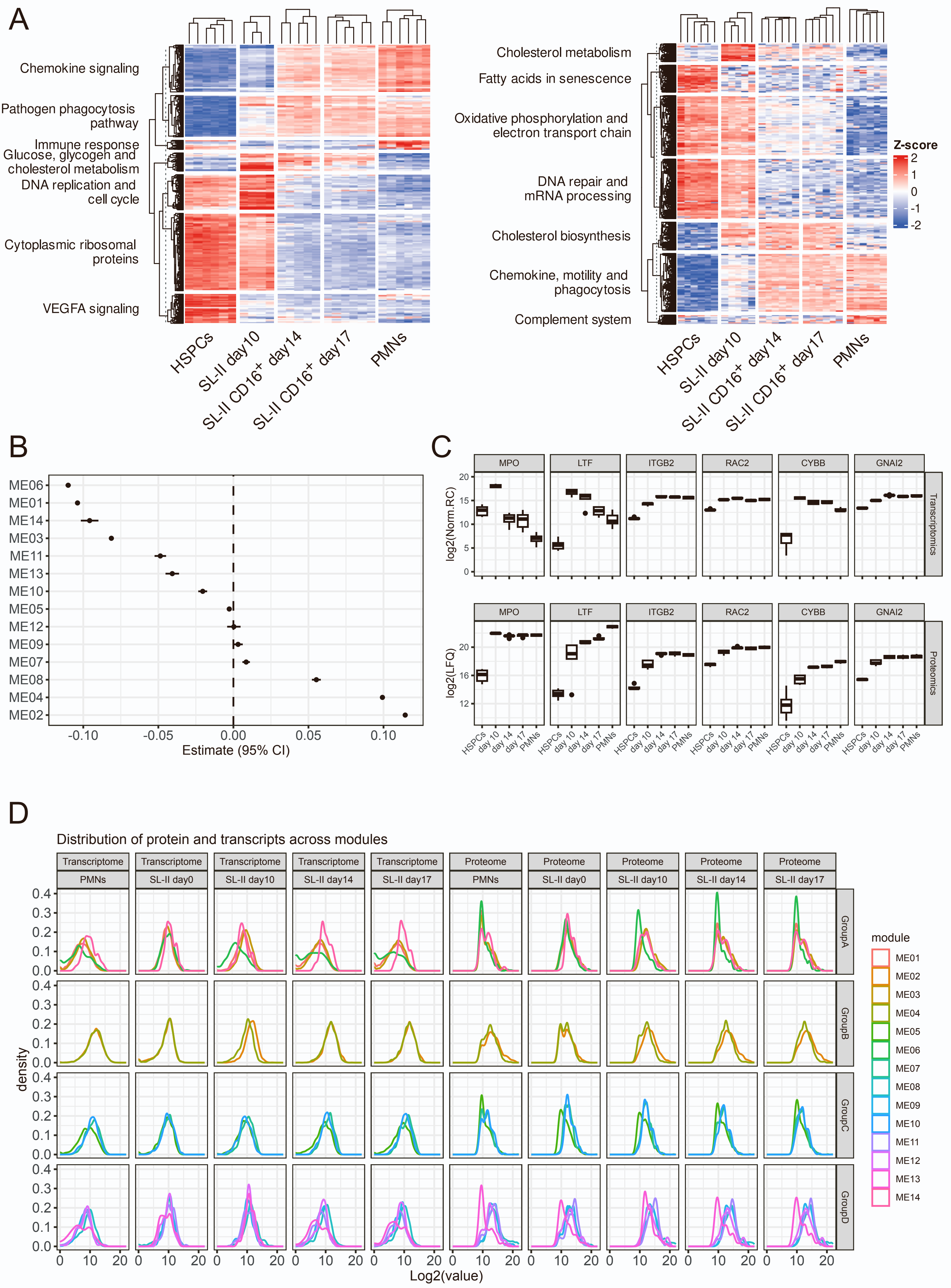
A
Chemokine signaling
Pathogen phagocytosis pathway
Immune response
Glucose, glycogen and cholesterol metabolism
DNA replication and cell cycle
Cytoplasmic ribosomal proteins
VEGFA signaling
HSPCs
SL-II day10
SL-II CD16+ day14
SL-II CD16+ day17
PMNs
Cholesterol metabolism
Fatty acids in senescence
Oxidative phosphorylation and electron transport chain
DNA repair and mRNA processing
Cholesterol biosynthesis
Chemokine, motility and phagocytosis
Complement system
Z-score
B
ME06
ME01
ME14
ME03
ME11
ME13
ME10
ME05
ME12
ME09
ME07
ME08
ME04
ME02
Estimate (95% CI)
C
MPO
LTF
ITGB2
RAC2
CYBB
GNAI2
log2(Norm.RC)
Transcriptomics
log2(LFQ)
Proteomics
D
Distribution of protein and transcripts across modules
Transcriptome
Proteome
PMNs
SL-II day0
SL-II day10
SL-II day14
SL-II day17
GroupA
GroupB
GroupC
GroupD
density
Log2(value)
module

Figure S5

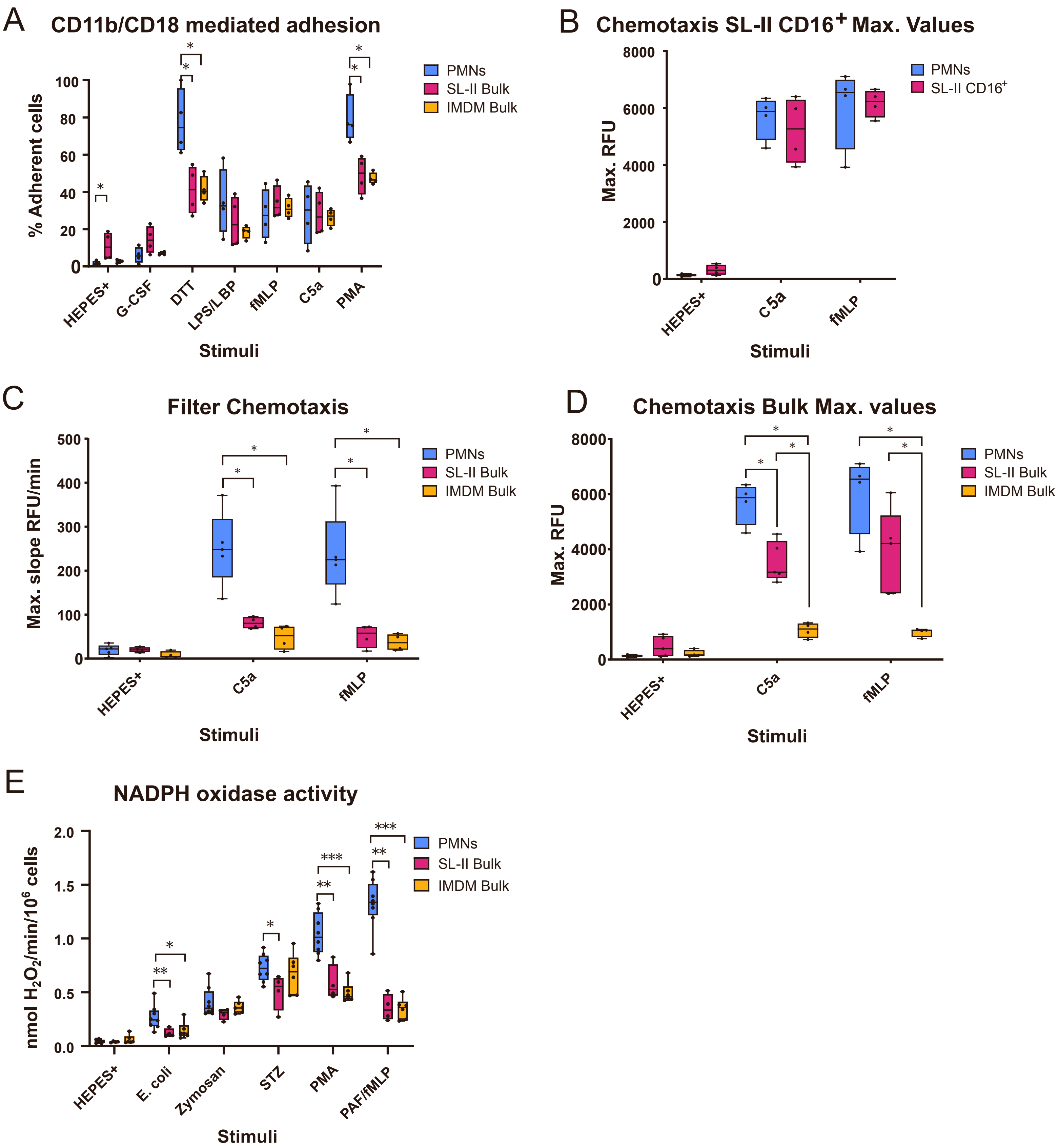
A
CD11b/CD18 mediated adhesion
% Adherent cells
0
20
40
60
80
100
HEPES+
G-CSF
DTT
LPS/LBP
fMLP
C5a
PMA
Stimuli
PMNs
SL-II Bulk
IMDM Bulk
*
B
Chemotaxis SL-II CD16+ Max. Values
Max. RFU
0
2000
4000
6000
8000
HEPES+
c5a
fMLP
Stimuli
PMNs
SL-II CD16+
C
Filter Chemotaxis
Max. slope RFU/min
0
100
200
300
400
500
HEPES+
C5a
fMLP
Stimuli
PMNs
SL-II Bulk
IMDM Bulk
D
Chemotaxis Bulk Max. values
Max. RFU
0
2000
4000
6000
8000
HEPES+
C5a
fMLP
Stimuli
PMNs
SL-II Bulk
IMDM Bulk
E
NADPH oxidase activity
nmol H2O2/min/10^6 cells
0.0
0.5
1.0
1.5
2.0
HEPES+
E. coli
Zymosan
STZ
PMA
PAF/fMLP
Stimuli
PMNs
SL-II Bulk
IMDM Bulk
**
***

Figure S6

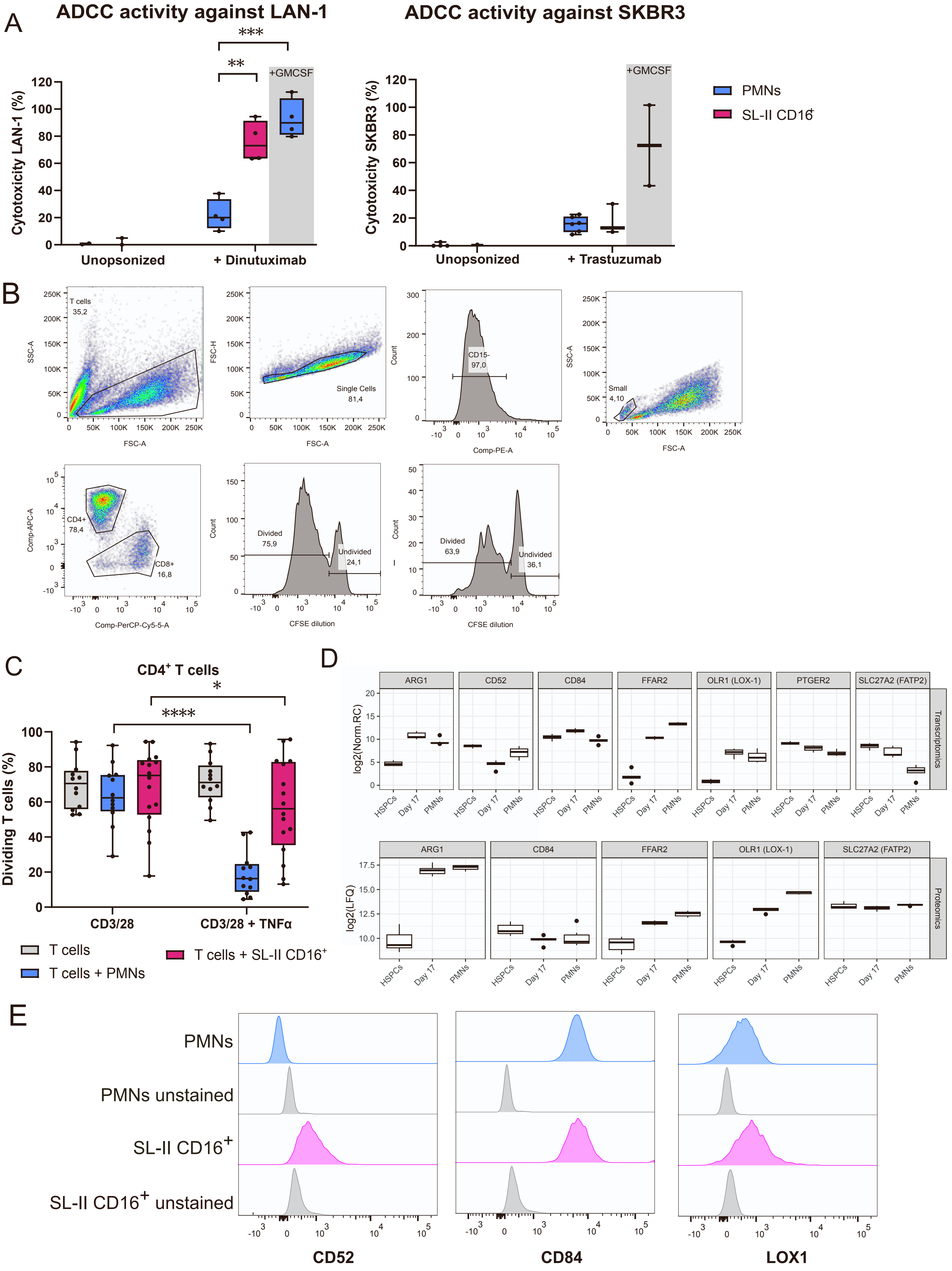

A
ADCC activity against LAN-1
ADCC activity against SKBR3
Cytotoxicity LAN-1 (%)
Cytotoxicity SKBR3 (%)
+GMCSF
Unopsonized
+ Dinutuximab
+ Trastuzumab
PMNs
SL-II CD16+
B
T cells 35,2
Single Cells 81,4
CD15- 97,0
Small 4,10
CD4+ 78,4
CD8+ 16,8
Divided 75,9
Undivided 24,1
Divided 63,9
Undivided 36,1
SSC-A
FSC-A
FSC-H
Count
Comp-PE-A
Comp-APC-A
Comp-PerCP-Cy5-5-A
CFSE dilution
C
CD4+ T cells
Dividing T cells (%)
CD3/28
CD3/28 + TNFα
T cells
T cells + PMNs
T cells + SL-II CD16+
D
ARG1
CD52
CD84
FFAR2
OLR1 (LOX-1)
PTGER2
SLC27A2 (FATP2)
Transcriptomics
Proteomics
log2(Norm.RC)
log2(LFQ)
HSPCs
Day 17
PMNs
E
PMNs
PMNs unstained
SL-II CD16+
SL-II CD16+ unstained
CD52
CD84
LOX1

Supplemental information titles and legends:

Figure S1: Culturing method for differentiation of CD34$^+$ HSPCs towards neutrophils using different culturing media. A. Culturing scheme for differentiating CD34$^+$ HSPCs towards neutrophils in a 17 day culture, including the experimental set-up of the study. **B.** Expression of CD11b/CD16 on SL-II cultured neutrophils of the CD16 enriched and CD16 depleted fraction after MACS isolation, respectively. **C.** Gating strategy for flow cytometry. First cells were gated, then single cells were selected followed by gating for CD15$^+$ cells and for CD11b$^+$CD16$^+$ neutrophils and the different fractions in Bulk SL-II day 17 cells based on the CD11b/CD16 expression, respectively. **D.** Flow cytometric analysis of surface markers SIGLEC9, EMR3, CD10 and CD101 expressed on Bulk and CD16$^+$ neutrophils cultured in SL-II medium (pink) or IMDM (orange) and PMNs (blue). Gray histograms show unstained controls.

Figure S2: Flow cytometric, transcriptomic and proteomic characterization of IMDM neutrophils compared to SL-II neutrophils and PMNs. A. Representative histograms of flow cytometric analysis of surface markers CD34, HLA-DR, CD66b, SIGLEC9, CD177 and CD10 expressed on CD16$^+$ neutrophils cultured in IMDM (orange) on day 0, 10, 14, 17 and PMNs (blue). Gray histograms show unstained controls. **B.** Heatmaps of transcriptome and proteome z-scores for HSPCs, IMDM Bulk day 14, SL-II Bulk day 14, SL-II CD16$^+$ day 14 and PMNs. All genes that were considered differentially abundant between any of these conditions and PMNs were included. **C.** Pearson correlation of all transcriptome and proteome samples that were included in this study.

Figure S3: Identification of proteins from exogenous sources. A. Protein-protein STRING-db interaction network for proteins for which no matching transcripts were identified in any of the SL-II samples or PMNs. Proteins are shown as nodes. Those that were enriched for platelet-plasma processes are highlighted in red. **B.** Scatterplot of intensity values for proteins without a matching transcript in SL-II CD16$^+$ day 17 and PMNs. Each individual dot represents one individual sample for which a valid value of the protein was found. The proportion of samples for which valid values were observed is shown as bar plots.

Figure S4: Differentiation of SL-II derived neutrophils. A. Heatmaps of transcriptome and proteome z-scores for HSPCs, SL-II Bulk day 10, SL-II CD16$^+$ day 14, SL-II CD16$^+$ day 17 and PMNs. All genes found to be differentially abundant between any condition and PMNs were included. **B.** Forest plot showing point estimates and 95% confidence interval (CI) for the association between time of differentiation (time as a continuous outcome) and z-scores for combining transcriptome and proteome together for each module separately. **C.** Boxplots showing the log2 normalized read counts and log2 LFQ for genes with high connectivity degree ranked by module membership along differentiation. **D.** Distribution of log2 normalized read counts and log2 LFQ for all modules collapsed in each of the four groups (A - D) define in figure 1. Data in (C) is represented as median and interquartile range of the abundance of transcripts and proteins for each independent differentiation stage and PMNs.

Figure S5: Both Bulk SL-II and IMDM neutrophils show decreased neutrophil effector functions compared to PMNs. A. CD11b/CD18-mediated adhesion assay of PMNs (blue), SL-II Bulk (pink) and IMDM Bulk (orange) to uncoated plastic plates (n=4). **B.** Maximum values

reached in the chemotaxis assay by PMNs (blue) and SL-II CD16$^+$ neutrophils (pink) (n=4). **C.** Chemotactic potential of fluorescently labelled PMNs (blue), SL-II Bulk (pink) and IMDM Bulk (orange) based on movement through filters with a pore size of 3 micron (n=4). **D.** Maximum values reached in the chemotaxis assay by PMNs (blue), SL-II Bulk (pink) and IMDM Bulk (orange) (n=4). **E.** NADPH oxidase assay to determine production of extracellular peroxide after addition of several stimuli for PMNs (blue), SL-II Bulk (pink) and IMDM Bulk (orange) (n=4). In the case of IMDM, killing, phagocytosis and ADCC activity were similar to those of SL-II culture neutrophils (data not shown). Data in (A), (B), (C), (D) and (E) is represented as median and interquartile range of each independent experiment. P values were calculated using Mann-Whitney U tests and labelled as *p < 0.05, **p < 0.01, ***p < 0.001 and ****p < 0.0001. N values represent the number of individual donor samples.

Figure S6: Analysis of MDSC activity exerted by PMNs and SL-II CD16$^+$ neutrophils *in vitro*. **A.** *In vitro* ADCC of LAN-1 cells unopsonized and opsonized (+Dinutuximab) (n=4) and SKBR3 cells unopsonized and opsonized (+Trastuzumab) (n=3) by PMNs (blue) and SL-II CD16$^+$ neutrophils (pink) and GM-CSF stimulated PMNs in the gray box in a 1:50 T:E ratio (n=3). **B.** Representative gating strategy assessing MDSC activity carried out by PMNs and SL-II CD16$^+$ neutrophils by gating on T cells, single cells and excluding all CD15-PE positive cells (PMNs and SL-II CD16$^+$ neutrophils). Subsequent gating on FSC^{low} (small) T cells and large T cells was based on FSC/SSC plots. Assessment of CD4$^+$ and CD8$^+$ T cell proliferation was performed using CD4-APC and CD8-PerCPCy5.5 separation, from which the CFSE dilution was analyzed separately. Representative gating strategy of n=12 with the addition of PMNs to T cell cultures and n=16 for SL-II CD16$^+$ neutrophils. **C.** *In vitro* MDSC activity measured by CD4$^+$ T cell proliferation. T

cells were stimulated with anti-CD3/CD28 antibodies to induce proliferation and co-cultured with either unstimulated or TNFα-stimulated PMNs (blue) (n=12) or SL-II $CD16^+$ neutrophils (pink) (n=16). After 4 days, T cell proliferation was assessed by CFSE dilution. Data in (A), (C) and (D) is represented as median and interquartile range of each independent experiment. P values were calculated using Mann-Whitney U tests and labelled as *p < 0.05, *p < 0.01, ***p < 0.001 and ****p < 0.0001. N values represent the number of individual donor samples.

Table S1: Percentage of positive cells and gMFI of 30 different surface markers measured using flow cytometry on SL-II and IMDM cultured neutrophils together with PMNs. The left column represents Bulk samples gated on $CD15^+$ cells, whereas the right column represents the most mature CD11b/CD16 fraction (n=3 for the white boxes, n=6 for the light gray boxes and n=10 for the dark gray boxes). N values represent the number of individual donor samples.

Table S2: Complete list of transcripts and proteins compared between SL-II $CD16^+$ cultured neutrophils and PMNs. Sub tabs were created for gene-protein subsets associated with high RNA and low protein abundance.

Table S3: Gene set enrichment analysis of up- and down-regulated transcripts and proteins that were obtained when comparing SL-II $CD16^+$ cultured neutrophils to PMNs. Biological processes were obtained either from Wiki-Pathways (*), Gene Onthology (**) or Reactome (***). All biological processes with an FDR < 0.05 were considered statistically significant.

Table S4: Protein-Protein interaction network of all proteins for which no transcript was observed in either PMNs or SL-II cultured neutrophils.

Table S5: Complete list of transcript/protein pairs contained in each one of the 14 modules identified using a WGCNA.

Table S6: Presence or absence of each of the transcript/protein pairs in Mitocarta3.0, Recon3D or the list of granule related proteins.

Table S7: Complete list of proteins which have been reported to be important for adhesion, chemotaxis, oxidative activity and phagocytosis, as well as intracellular signaling in human neutrophils.

Table S8: Curated list of 111 neutrophil granule proteins.

Video S1: Representative video of the taxiscan assay showing the unstimulated chemotactic capability of PMNs (n=4). HEPES+ medium was used as a negative control. N values represent the number of individual donor samples.

Video S2: Representative video of the taxiscan assay showing the unstimulated chemotactic capability of SL-II $CD16^+$ cultured neutrophils (n=4). HEPES+ medium was used as a negative control. N values represent the number of individual donor samples.

Video S3: Representative video of the taxiscan assay showing the chemotactic capability of PMNs towards fMLP used as chemoattractant (n=4). N values represent the number of individual donor samples.

Video S4: Representative video of the taxiscan assay showing the chemotactic capability of SL-II $CD16^+$ cultured neutrophils towards fMLP used as chemoattractant (n=3). N values represent the number of individual donor samples.

Video S5: Representative video of SL-II $CD16^+$ cultured neutrophils performing trogocytosis of T cells (n=5). Here, we display a neutrophil containing a piece of T cell membrane inside

the cytoplasm. The SL-II $CD16^+$ cultured neutrophil is shown in orange and the T cell membrane is shown in pink. N values represent the number of individual donor samples.

Table S1.

		PMNs	SL-II	IMDM
All Neutrophils	CD15+ (%)	99	97	53
	CD15+ (gMFI)	10528	4643	3101
	CD33 (%)	97	87	92
	CD33 (gMFI)	1250	2614	9566
	CD34 (%)	10	3	1
	CD34 (gMFI)	27	19	-34
	CD45 (%)	99	98	93
	CD45 (gMFI)	2781	2617	3793
	CD11b (%)	98	83	75
	CD11b (gMFI)	11470	3753	4585
	CD16 (%)	98	87	59
	CD16(gMFI)	100812	5138	1213
	CD10 (%)	96	39	2
	CD10 (gMFI)	1947	414	-4
	EMR3 (%)	99	55	90
	EMR3 (gMFI)	2120	642	9079
	Siglec9 (%)	95	91	64
	Siglec9 (gMFI)	4443	4902	2299
	CD177 (%)	64	79	40
	CD177 (gMFI)	1715	8592	1709
	CD66b (%)	99	93	68
	CD66b (gMFI)	5346	4970	2083
	CD101 (%)	99	32	17
	CD101 (gMFI)	4142	367	423
	CD63 (%)	24	25	34
	CD63 (gMFI)	226	249	581
	LOX1 (%)	58	67	89
	LOX1 (gMFI)	583	654	3627
	HLA DR (%)	0	1	14
	HLA DR (gMFI)	0	1	242
	CD29 (%)	23	69	86
	CD29 (gMFI)	175	680	1921
	CD14 (%)	25	36	91
	CD14 (gMFI)	203	342	4398
	FPR1 (%)	37	76	90
	FPR1 (gMFI)	322	1047	8426
	CD69 (%)	3	1	21
	CD69 (gMFI)	0	0	291
	CD61 (%)	4	0	-1
	CD61 (gMFI)	0	0	-39

	CD88 (%)	94	74	90
	CD88 (gMFI)	1587	1151	2459
	CD62L (%)	98	40	40
	CD62L (gMFI)	10085	695	1532
	CD64 (%)	1	27	89
	CD64 (gMFI)	0	373	4723
	CD32 (%)	98	97	88
	CD32 (gMFI)	9726	3713	6924
	CD41a (%)	62	0	1
	CD41a (gMFI)	1048	0	14
	CD36 (%)	0	0	-1
	CD36 (gMFI)	0	0	-13
	PDL1 (%)	47	41	47
	PDL1 (gMFI)	270	479	357
	CD52 (%)	3	72	88
	CD52 (gMFI)	0	1178	19125
	CD84 (%)	94	97	65
	CD84 (gMFI)	3960	4642	6654

		PMNs	SL-II	IMDM
CD16high population	CD16 (%)	100	100	96
	CD16(gMFI)	140803	30303	11773
	CD10 (%)	98	62	33
	CD10 (gMFI)	2244	654	388
	EMR3 (%)	99	47	91
	EMR3 (gMFI)	2120	459	11314
	Siglec9 (%)	97	98	89
	Siglec9 (gMFI)	5746	13078	11805
	CD177 (%)	65	93	80
	CD177 (gMFI)	1653	25824	5001
	CD66b (%)	99	95	85
	CD66b (gMFI)	5213	3878	2494
	CD101 (%)	99	54	37
	CD101 (gMFI)	4129	814	1066
	CD63 (%)	24	27	40
	CD63 (gMFI)	173	267	788
	LOX1 (%)	58	67	92
	LOX1 (gMFI)	616	632	7041
	HLA DR (%)	0	0	33
	HLA DR (gMFI)	0	0	944

	CD29 (%)	21	71	88
	CD29 (gMFI)	125	532	3955
	CD14 (%)	29	59	95
	CD14 (gMFI)	280	567	29553
	FPR1 (%)	33	61	91
	FPR1 (gMFI)	322	732	12627
	CD69 (%)	52	50	67
	CD69 (gMFI)	21780	16996	2346
	CD61 (%)	3	0	35
	CD61 (gMFI)	0	0	574
	CD88 (%)	96	96	94
	CD88 (gMFI)	1673	1976	6433
	CD62L (%)	98	75	58
	CD62L (gMFI)	10645	1702	2144
	CD64 (%)	0	15	91
	CD64 (gMFI)	0	221	6074
	CD32 (%)	98	98	91
	CD32 (gMFI)	10305	6732	30066
	CD41a (%)	61	0	20
	CD41a (gMFI)	1016	0	292
	CD36 (%)	0	0	36
	CD36 (gMFI)	0	0	579
	PDL1 (%)	46	56	68
	PDL1 (gMFI)	269	687	1342
	CD52 (%)	0	69	89
	CD52 (gMFI)	0	829	23339
	CD84 (%)	93	98	67
	CD84 (gMFI)	3791	4990	25684

Table S7.

Hugo symbol	Curated function
CXCR1	Chemotaxis
CXCR2	Chemotaxis
C5AR1	Chemotaxis
PTAFR	Chemotaxis
LTB4R	Chemotaxis
FPR1	Chemotaxis
FPR2	Chemotaxis
FPR3	Chemotaxis
TLR1	Pathogen recognition
TLR2	Pathogen recognition
TLR4	Pathogen recognition
TLR5	Pathogen recognition
TLR6	Pathogen recognition
TLR8	Pathogen recognition
FCGR1A	Phagocytosis
FCGR2A	Phagocytosis
FCGR3B	Phagocytosis
CR1	Phagocytosis
ITGAL	Adhesion
ITGAM	Adhesion
ITGAX	Adhesion
ITGB2	Adhesion
TLN1	Adhesion
FERMT3	Adhesion
APBB1IP	Adhesion
RAP1A	Adhesion
PIP5K1C	Adhesion
ILK	Adhesion
PRKCA	Signaling
SYK	Signaling
HCK	Signaling
LYN	Signaling
FGR	Signaling
RACK1	Signaling
FGD4	Signaling
CAPN1	Phagocytosis
PARVG	Adhesion
ELMO1	Phagocytosis
DOCK2	Signaling
RHOG	Signaling

PTK2	Signaling
PLCB2	Signaling
PIK3CG	Signaling
GNAI2	Signaling
RGS5	Signaling
RAC2	Signaling
PREX1	Signaling
ARHGEF6	Signaling
TIAM1	Signaling
PXN	Adhesion
VCL	Adhesion
ADAP1	Signaling
PLCG2	Signaling
ARHGAP25	Signaling
VAV1	Signaling
VAV3	Signaling
CYBC	Oxidase activity
MPO	Oxidase activity
CYBB	Oxidase activity
CYBA	Oxidase activity
NCF1	Oxidase activity
NCF2	Oxidase activity
NCF4	Oxidase activity
ACTR2	Chemotaxis
ACTR3	Chemotaxis
ARPC1B	Chemotaxis
ARPC2	Chemotaxis
ARPC3	Chemotaxis
ARPC4	Chemotaxis
ARPC5	Chemotaxis
WASF2	Chemotaxis
BRK1	Chemotaxis
ABI1	Chemotaxis
NCKAP1L	Chemotaxis
CYFIP1	Chemotaxis

Table S8.

Granule reported in Rørvig, et al, 2013	Protein name as reported in the original dataset (Rørvig, et al, 2013)	Hugo symbol
AG	1,4-beta-N-acetylmuramidase C	LYZ
AG	Alpha-1-antichymotrypsin	SERPINA3
AG	Alpha-D-galactosidase A	GLA
AG	Alpha-L-fucosidase I	FUCA1
AG	Alpha-L-fucoside fucohydrolase 2	FUCA2
AG	Arylsulfatase A	ARSA
AG	Arylsulfatase B	ARSB
AG	Azurocidin	AZU1
AG	Bactericidal permeability-increasing protein	BPI
AG	Beta-galactosidase	GLB1
AG	Beta-glucuronidase	GUSB
AG	Beta-hexosaminidase alpha chain	HEXA
AG	Beta-hexosaminidase subunit beta	HEXB
AG	Beta-mannosidase	MANBA
AG	cathepsin A isoform a precursor	CTSA
AG	Cathepsin C	CTSC
AG	Cathepsin G	CTSG
AG	CD107 antigen-like family member A	LAMP1
AG	CD107 antigen-like family member B	LAMP2
AG	CD63 antigen	CD63
AG	Ferritin light chain	FTL
AG	Grancalcin	GCA
AG	Granulin A	GRN
AG	Leukocyte proteinase 3	PRTN3
AG	Lysosomal alpha-mannosidase	MAN2B1
AG	Myeloperoxidase	MPO
AG	Neutrophil defensin 1	DEFA1
AG	Neutrophil elastase	ELANE
AG	Placenta-specific gene 8 protein	PLAC8
SG	Antileukoproteinase	SLPI
SG	C3a anaphylatoxin chemotactic receptor	C3AR1
SG	Cathelicidin antimicrobial peptide precursor	CAMP
SG	CD11 antigen-like family member A	ITGAL
SG	CD20 antigen-like protein	MS4A3
SG	CD47 antigen isoform 3 precursor	CD47
SG	CD59 antigen, complement regulatory protein	CD59
SG	Chitinase-1	CHIT1

SG	Chitinase-3-like protein 1	CHI3L1
SG	Choline phosphatase 1	PLD1
SG	C-type lectin domain family 12 member A	CLEC12A
SG	C-type lectin domain family 4 member D	CLEC4D
SG	C-type lectin domain family 5 member A	CLEC5A
SG	C-type lectin domain family 8 member A	OLR1
SG	Cysteine-rich secretory protein 3	CRISP3
SG	Cytochrome b(558) alpha chain	CYBA
SG	Dedicator of cytokinesis protein 2	DOCK2
SG	Defensin, alpha 4	DEFA4
SG	Eosinophil major basic protein homolog	PRG3
SG	G-protein coupled receptor 84	GPR84
SG	Haptoglobin	HP
SG	Immunoglobulin alpha Fc receptor	FCAR
SG	Integrin, alpha M (Complement component 3 receptor 3 subunit)	ITGAM
SG	Lactoferrin	LTF
SG	Leucine-rich alpha-2-glycoprotein	LRG1
SG	Leucine-rich repeat-containing protein 7	LRRC7
SG	Leukocyte-associated immunoglobulin-like receptor 1	LAIR1
SG	Matrix metalloproteinase-8	MMP8
SG	Metalloproteinase inhibitor 2	TIMP2
SG	Myeloid cell surface antigen CD33	CD33
SG	Neutrophil gelatinase-associated lipocalin	LCN2
SG	Olfactomedin-4	OLFM4
SG	Bone marrow proteoglycan	OSCAR
SG	Transcobalamin I	TCN1
SG	Urokinase-type plasminogen activator	PLAU
SG	Vesicle-associated membrane protein 8	VAMP8
GG	Beta-2-microglobulin	B2M
GG	CD177 antigen	CD177
GG	Matrix metalloproteinase-9	MMP9
GG	Serine protease 3	PRSS3
GG	Bone marrow proteoglycan	PRG2
GG	C3b/C4b receptor	CR1
GG	Cathepsin B	CTSB
GG	Cathepsin D	CTSD
GG	Cathepsin H	CTSH
GG	Cathepsin P	CTSZ
GG	Cathepsin S	CTSS
GG	CD11 antigen-like family member C	ITGAX

GG	CD300 antigen-like family member A	CD300A
GG	CD33 antigen-like 2	
GG	CD85 antigen-like family member D	LILRB2
GG	CD85 antigen-like family member E	
GG	Complement component C1q receptor	CD93
GG	Cystatin-3	CST3
GG	Cystatin-B	CSTB
GG	Cysteine-rich secretory protein 11	CRISPLD2
GG	Ferritin heavy chain	FTH1
GG	Fibrinogen-like protein 2	FGL2
GG	Ficolin-1	FCN1
GG	Integrin beta	ITGB2
GG	Integrin beta-2	ITGB2
GG	N-formyl peptide receptor 2	FPR2
GG	Platelet-activating factor acetylhydrolase IB subunit beta	PAFAH1B2
GG	Semaphorin-4A	SEMA4A
SG	Pentaxin-related protein PTX3	PTX3
SV	Annexin A1	ANXA1
SV	Annexin A11	ANXA11
SV	Annexin A3	ANXA3
SV	Annexin A5	ANXA5
SV	annexin IV	ANXA4
SV	Cystatin-A	CSTA
SV	Galectin-3	LGALS3
SV	Macrophage migration inhibitory factor	MIF
SV	Matrix metalloproteinase-25	MMP25
SV	Osteoclast-stimulating factor 1	OSTF1
SV	CD85 antigen-like family member A	LILRB3
SV	CD97 antigen	ADGRE5
SV	Fc-gamma RIII-beta	FCGR3B
SV	Monocyte differentiation antigen CD14	CD14
SV	Opioid growth factor receptor	OGFR
SV	P2X purinoceptor 1	P2RX1
SV	Toll/interleukin-1 receptor-like protein 4	TLR4
